# Clinical Performance of a Fully Integrated Sample-to-Answer Multiplex PCR Respiratory Panel: A Multicenter Evaluation Using Prospective, Archived and Contrived Nasopharyngeal Specimens

**DOI:** 10.3390/diagnostics16213541

**Published:** 2026-10-28

**Authors:** Chi-Sheng Tai, Hsing-Yi Chung, Tai-Han Lin, Chih-Kai Chang, Cherng-Lih Perng, Yifan Lin, Uanting Tsai, Matthew Beltrami, Emily Zamot, Hung-Sheng Shang, Ming-Jr Jian

**Affiliations:** 1 Graduate Institute of Medical Science, National Defense Medical University, Taipei City 11490, Taiwan; eric7142@gmail.com; 2 Division of Clinical Pathology, Department of Pathology, Tri-Service General Hospital, National Defense Medical University, Taipei City 11490, Taiwan; cindyft12@gmail.com (H.-Y.C.); garlicbun84@gmail.com (T.-H.L.); mblkaiser@gmail.com (C.-K.C.); ponchenli@gmail.com (C.-L.P.); 3 LabTurbo Biotech US CLIA High-Complexity Laboratory, LabTurbo Biotech LLC, Princeton, NJ 08540, USA; elin@labturbo.com (Y.L.); ttsai@labturbo.com (U.T.); mbeltrami@labturbo.com (M.B.); ezamot@labturbo.com (E.Z.); 4 Graduate Institute of Pathology and Parasitology, National Defense Medical University, Taipei City 11490, Taiwan

**Keywords:** multiplex PCR, syndromic testing, molecular diagnostics, diagnostic stewardship, respiratory pathogens, sample-to-answer, *Mycoplasma pneumoniae*, *Bordetella pertussis*, positive percent agreement, nasopharyngeal swab

## Abstract

**Background:** Most syndromic respiratory PCR panels process one specimen per cartridge and instrument module. **Objectives:** We evaluated the agreement of a fully integrated, batch-capable sample-to-answer 22-target respiratory panel with BioFire FilmArray Respiratory Panel 2.1. **Methods:** Nasopharyngeal swabs were collected prospectively from symptomatic individuals aged ≥ 2 years at three US clinical sites between December 2025 and May 2026. Investigational and comparator testing were performed in separate, mutually blinded CLIA-certified laboratories; discordant specimens were retested with a third molecular assay, reported descriptively. Archived (*n* = 240) and contrived (*n* = 200) specimens for low-prevalence targets were analyzed separately. Positive and negative percent agreement (PPA, NPA) were reported with Wilson score 95% confidence intervals (CI). **Results:** Of 1501 participants enrolled, 1482 specimens yielded paired valid results; final invalid rates after repeat testing were 0.07% and 0.13%, respectively. Twelve targets met all prespecified prospective acceptance criteria (PPA 91.5–100%). Seven targets had PPA point estimates of 90.0–100% but lower 95% CI bounds below 80%, and three had no prospective positives. NPA was 99.6–100% for all 22 targets. **Conclusions:** The system showed high agreement with the comparator for targets well represented in prospective specimens. For the remaining ten targets, prospective evidence was insufficient to confirm the acceptance criteria; archived and contrived results provide supportive evidence of detection but do not change this prospective classification.

## 1. Introduction

Acute respiratory infections remain one of the most frequent reasons for outpatient consultation and hospital admission worldwide, with lower respiratory tract infections ranking among the leading infectious causes of death across all age groups [1,2]. The clinical syndromes caused by influenza viruses, the respiratory syncytial virus (RSV), SARS-CoV-2, human metapneumovirus, seasonal coronaviruses, rhinovirus, and atypical bacterial pathogens overlap extensively; consequently, history and examination alone cannot establish an etiology [3,4]. Additionally, since the reordering of respiratory virus circulation following the COVID-19 pandemic, several of these infectious agents have now co-circulated outside their historical seasons, further reducing the diagnostic value of epidemiological reasoning at the bedside [5].

Syndromic multiplex nucleic acid amplification panels investigate a broad set of respiratory targets in a single specimen during a single run. Evaluations of widely deployed cartridge-based systems, including multicenter studies, have reported high agreement with molecular reference methods and have established these platforms in routine practice [6,7,8,9,10,11]. However, the actual benefits of such testing remain debatable. A 2024 systematic review and meta-analysis of 11 randomized trials in emergency departments found no association between rapid respiratory virus testing and antibiotic use, emergency department length of stay, return visits, or hospitalization. Rather, testing was associated with greater use of influenza antivirals and with modest reductions in chest radiography and blood testing, although the absolute effects were small [12]. An earlier meta-analysis of point-of-care influenza testing in ambulatory care reached a similar conclusion regarding antibiotic prescription [13], while individual randomized and observational studies have reported a shorter time to antiviral treatment, appropriate isolation, and reduced ancillary testing [14,15]. Although current professional guidance recommends molecular rather than antigen detection when a result will influence management [16,17], which patients benefit from a broad syndromic panel remains unknown.

Furthermore, most cartridge-based systems process one specimen per instrument module for the duration of the run, so that capacity increases mainly with the number of modules. A platform that combines automated nucleic acid extraction with real-time PCR in one closed system, while allowing several specimens to be processed in a batch, offers a different workflow. Whether such a design translates into higher throughput, shorter turnaround time or lower cost depends on local volumes and staffing and must be measured directly; these outcomes were not assessed in this study.

The LabTurbo AIO Respiratory Panel PCR (RPP) Test System integrates automated nucleic acid extraction and multiplex real-time PCR for 22 respiratory targets within a single instrument, requiring no separate extraction step or manual transfer of purified nucleic acids. However, prospective specimens collected across the intended use settings, and a strategy for targets whose seasonal prevalence is too low to support a precise agreement estimate from a prospective collection alone, are required to characterize the agreement of such a system with an established panel [6,18]. Therefore, we aimed to evaluate the agreement of the RPP Test System with BioFire FilmArray Respiratory Panel 2.1 in symptomatic patients at three geographically separated US clinical sites, and to report archived and contrived specimen results separately for the least prevalent targets. The study was designed as an agreement study against prespecified acceptance criteria and not as a formal equivalence or non-inferiority study.

## 2. Materials and Methods

### 2.1. Study Design and Ethical Oversight

The clinical study was a non-interventional, multi-center diagnostic agreement study of the LabTurbo AIO RPP Test System under IRB-approved protocol LT-002, in which the investigational and comparator methods were performed in separate laboratories by personnel blinded to the results of the other method (Section 2.5). Nasopharyngeal swab (NPS) specimens in UTM were collected from symptomatic individuals at multiple clinical sites. The primary objective of this study was to compare the qualitative results of the investigational system with those of the comparator method for a 22-target panel. Acceptance criteria were prespecified before any testing: PPA of at least 90% with a lower 95% confidence bound of at least 80.0% for every target, PPA of at least 95% with the same lower bound for SARS-CoV-2, and NPA of at least 95% with a lower 95% confidence bound of at least 80.0% for every target. The protocol further specified that targets for which prospective collection would yield insufficient positive results would be supplemented with archived or contrived specimens. Although the protocol stated its objective in the regulatory terms used for premarket submissions, the study was designed statistically to estimate agreement against these prespecified per-target criteria; it was not designed as an equivalence or non-inferiority comparison, and no equivalence margin or comparative hypothesis test was specified.

This study is reported following the STARD 2015 recommendations for diagnostic accuracy studies [19] and was conducted in accordance with the principles of the Declaration of Helsinki [20]. The master protocol, informed consent and assent templates, and use of residual specimens under a waiver of informed consent were reviewed and approved by the central institutional review board, with site-specific approvals obtained by each participating organization as applicable. Prospective specimens were obtained through all-comers enrollment under informed consent or as deidentified residual specimens from standard-of-care respiratory pathogen testing under an IRB-approved waiver of informed consent. The prospective clinical study was supplemented with frozen archived specimens obtained from geographically diverse clinical laboratories in the United States and internationally. Individual collection sites and testing laboratories were identified throughout this report by facility type and state only; institutional names were withheld at the request of the participating organizations. Because the study was non-interventional, it was not registered in a public clinical trial registry. The full protocol (LT-002, version 2.0) is available from the corresponding authors upon reasonable request, with the names of participating institutions replaced by coded identifiers (Data Availability Statement); its provisions relevant to this report are described in Section 2.1, Section 2.2, Section 2.3, Section 2.4, Section 2.5, Section 2.6, Section 2.7, Section 2.8 and Section 2.9.

### 2.2. Sites, Participants and Specimens

Nasopharyngeal swab (NPS) specimens were prospectively collected from patients at three US clinical sites selected to span the intended use settings and provide geographic and demographic diversity: a pediatric primary care practice in New Jersey (Site 1), an urgent care center in California (Site 2), and a hospital-based service in New York State (Site 3). The specimen accrual ran from December 2025 to May 2026, spanning a single Northern Hemisphere respiratory season. All the sites followed the same protocol and were subjected to the same monitoring process (Table 1).

**Table 1:** Specimen sets, collection settings and testing arrangement. Institutional names are withheld at the request of the participating organizations.

Specimen Set	Setting and Origin	n	Investigational Testing Laboratory	Comparator (Testing Laboratory)
Prospective	Site 1: pediatric primary care practice, New Jersey	499	Laboratory B: CLIA laboratory, Pennsylvania	FilmArray RP2.1 (Laboratory A)
	Site 2: urgent care center, California	486	Laboratory C: CLIA laboratory, California ^a^	FilmArray RP2.1 (Laboratory A)
	Site 3: hospital-based service, New York State	500	Laboratory A: sponsor’s CLIA laboratory, New Jersey	FilmArray RP2.1 (Laboratory D: Site 3 hospital laboratory)
Archived	Frozen banked NPS specimens from clinical laboratories in the United States and internationally, selected on historical result	240	Laboratories A, B and C, distributed evenly	FilmArray RP2.1 (Laboratories A and D)
Contrived	Representative strains of four low-prevalence targets spiked into negative clinical specimen pools at 2×, 5× and 100× LoD (Table S8)	200	Laboratories A, B and C	Known composition

NPS, nasopharyngeal swab; LoD, limit of detection. Prospective counts were the specimens accepted for testing; 1482 of 1485 specimens yielded valid paired results (Figure 1). Laboratory A, operated by the sponsor, performed investigational testing for Site 3 and comparator testing for Sites 1 and 2; no specimen was tested by both methods in the same laboratory. Discordant specimens were retested with QIAstat-Dx Respiratory Panel Plus at a fifth, independent CLIA-certified laboratory in California (Laboratory E). ^a^ Under common ownership with the contract research organization that managed the study.

**Figure 1:**
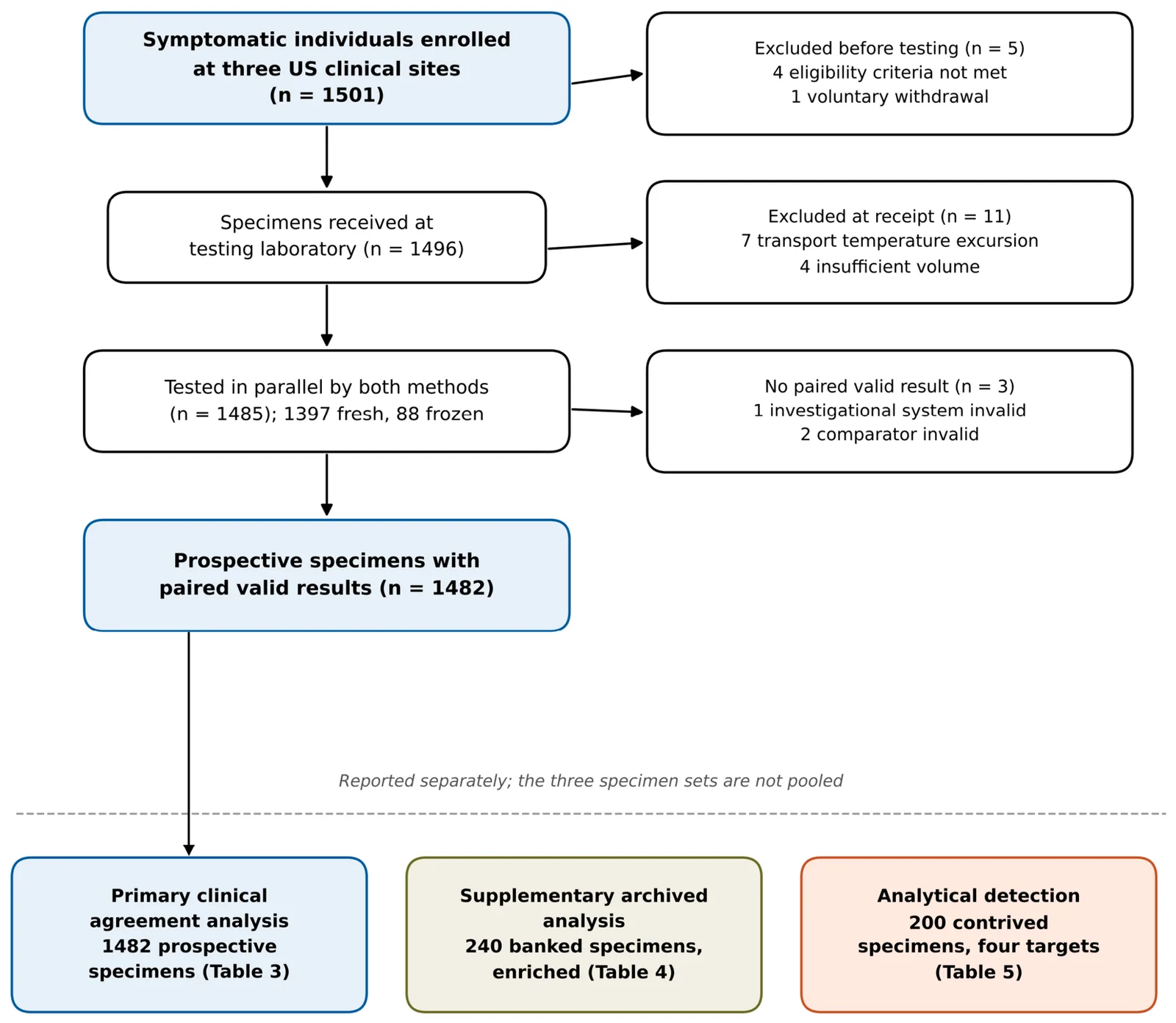
Disposition of specimens. The prospective cascade supports the primary clinical agreement analysis. The archived and contrived sets are shown as separate branches and were not pooled with the prospective specimens, for different reasons. Archived specimens were clinical specimens selected on their historical result and enriched for the targets they carry, so they do not represent an unselected clinical population; contrived specimens were prepared by spiking negative clinical matrix and were used to assess analytical detection of four targets that clinical collection did not represent. Specimens with a discordant result between the investigational system and the comparator were additionally retested using a third molecular assay for discordance resolution at an independent laboratory; adjudication did not alter the composition of any analysis set.

The specimens were obtained via one of two routes. Prospectively consented specimens were collected by a healthcare professional from patients aged ≥ 2 years in whom a provider suspected respiratory infection on the basis of fever, cough, sore throat, rhinorrhea, myalgia, headache, chills or fatigue. Patients with life-threatening illnesses, active nasal trauma such as epistaxis, or those who had received a nasal rinse, wash, or aspirate within the preceding hour were excluded. Alternatively, residual NPS specimens remaining after standard-of-care respiratory testing ordered by a physician were enrolled as consecutive all-comers under a waiver of informed consent, provided that the specimen had been collected in Copan Universal Transport Medium, with at least 1.5 mL remaining, age and sex were recorded, and the specimen was de-identified before transfer.

Specimens held at 2–8 °C and tested within 72 h of collection were designated as category I (fresh) specimen; specimens frozen below −20 °C because testing would be delayed beyond 72 h were designated category II (frozen) specimen. At receipt, specimens were rejected if they had been refrigerated for more than 72 h, frozen for more than 7 days, subjected to more than two freeze–thaw cycles, or were contaminated or deteriorated in the opinion of the investigator.

### 2.3. Investigational System

The LabTurbo AIO Respiratory Panel PCR Test System (LabTurbo Biotech LLC, Princeton, NJ, USA) performs automated nucleic acid extraction and multiplex real-time PCR using a single closed instrument without a separate extraction platform or manual transfer of the eluate. The panel reports the following 22 targets: adenovirus; human coronaviruses 229E (HCoV-229E), HKU1 (HCoV-HKU1), NL63 (HCoV-NL63) and OC43 (HCoV-OC43); SARS-CoV-2; human metapneumovirus; human rhinovirus/enterovirus; influenza A virus with subtyping for H1, H3, and H1-2009; influenza B virus; parainfluenza viruses 1 to 4; RSV; and bacterial targets *Bordetella parapertussis*, *Bordetella pertussis*, *Chlamydia pneumoniae* and *Mycoplasma pneumoniae*. Testing was performed according to the manufacturer’s instructions. Ten operators performed investigational testing across the three laboratories, all of whom had received system training beforehand; their characteristics are described in Section 2.7.

### 2.4. Comparator Method and Discordance Resolution

BioFire FilmArray Respiratory Panel 2.1 (bioMérieux, Salt Lake City, UT, USA), a multiplex molecular respiratory panel, was used according to the manufacturer’s instructions as the comparator method. Aliquots of each specimen were tested by the investigational system and comparator in separate, independent CLIA-certified laboratories: investigational testing at three laboratories located in New Jersey, Pennsylvania, and California, and comparator testing at a central laboratory in New Jersey and at the laboratory of the New York State hospital site. The sponsor’s CLIA-certified laboratory in New Jersey served as the investigational testing laboratory for Site 3 and as the central comparator laboratory for Sites 1 and 2; the assignment of specimens to laboratories and the measures used to keep the two methods blinded are described in Section 2.5.

Specimens with discordant results for any target were retested using QIAstat-Dx Respiratory Panel Plus (QIAGEN, Hilden, Germany), a multiplex molecular respiratory panel, for discordance resolution at an independent CLIA-certified laboratory in California, blinded to the original results. Retesting necessarily took place after both original results were available, so the material tested had been stored for longer than at the original tests. Adjudication results were reported descriptively and were not used to reclassify discordances in the primary agreement analysis, which retained the comparator as the reference. Because the third assay was applied only to discordant specimens, it was not treated as a reference standard, and its results were not used to attribute error to either method.

### 2.5. Role of the Sponsor, Laboratory Assignment and Blinding

The sponsor, the manufacturer of the investigational system, funded the study, wrote the protocol and supplied the investigational system. A contract research organization (CRO) managed the study, including site coordination, monitoring and the electronic data capture system. Four authors (Y.L., U.T., M.B. and E.Z.) are employees of the sponsor; their contributions are listed in the Author Contributions. The statistical analysis, the interpretation of the results and the drafting of the manuscript were performed by the academic authors.

Each collection site was assigned to one investigational testing laboratory and one comparator testing laboratory for the duration of the study (Table 1), and for every specimen the two methods were performed in different laboratories. The study records do not state further criteria for these assignments. The sponsor’s laboratory (Laboratory A) performed investigational testing only for Site 3 and comparator testing only for Sites 1 and 2; it therefore never tested the same specimen with both methods. Investigational testing for Sites 1 and 2 was performed at CLIA-certified laboratories in Pennsylvania (Laboratory B) and California (Laboratory C), and comparator testing for Site 3 at the hospital laboratory of that site (Laboratory D). Laboratory C is a separate legal entity under common ownership with the CRO, which engaged it to perform investigational testing for Site 2 under the oversight of its own laboratory director. Archived and contrived specimens were randomized and distributed evenly across the three investigational testing laboratories; comparator testing of archived specimens was performed at Laboratories A and D, and according to the sponsor’s records no laboratory tested the same archived specimen with both methods.

As specified in the protocol, specimens were aliquoted into tubes labelled with study identifiers that allowed the origin of each specimen to be traced without patient identifiers, and the second aliquot of each specimen was analyzed independently by a different trained technician with the comparator method. Results were entered by laboratory personnel directly into case report forms in an electronic data capture system (Greenlight Guru) managed by the CRO, compliant with 21 CFR Part 11 and with a built-in audit trail. Personnel at each testing laboratory could access only their own laboratory’s results and could not view the results of the other method. Authorized CRO personnel could view the results of both methods as they were entered but did not disclose results from one laboratory to testing personnel at another. Sponsor personnel had no system access to the results of both methods; testing personnel at Laboratory A could see only Laboratory A’s results, which for any given specimen came from one method only. The historical results of archived specimens were known only to the CRO, and comparator testing personnel were blinded to them. Discordant specimens were retested at a fifth, independent laboratory, which reported results blinded to the original outcomes.

The academic authors received the clinical study report, which tabulates the per-target results for every specimen set, and independently recalculated all agreement estimates, confidence intervals and acceptance-criterion classifications from these counts; the analysis code is provided as Supplementary File S1. They did not have access to the line-level data or instrument output during the analysis, so their verification was limited to the per-target counts reported in the clinical study report. The sponsor has since given standing written authorization for the de-identified line-level dataset to be provided (Data Availability Statement); however, the academic authors did not re-derive the per-target counts from line-level data for this report, and instrument output was not examined.

### 2.6. Archived and Contrived Specimens

Targets encountered infrequently during prospective collection were analyzed using 240 frozen, archived NPS specimens obtained from clinical laboratories in the United States and internationally and selected based solely on their historical qualitative results. Twenty known-negative specimens were interleaved for blinding, and the entire set was randomized before testing and distributed evenly across the three testing laboratories. The microbial content of the archived specimens was confirmed using the same comparator method used for the prospective specimens.

Four targets remained insufficiently represented after prospective and archived testing: influenza A virus H1, *B. parapertussis*, *B. pertussis* and *C. pneumoniae*. These targets were analyzed using 50 contrived positive specimens per target, prepared by spiking a representative strain into pools of confirmed negative clinical specimens at 2× (n = 30), 5× (n = 10), and 100× (n = 10) the limit of detection, yielding 200 contrived specimens in total. They were randomized and tested blindly across the three testing laboratories. The strains, their sources and the concentrations used are listed in Table S8. The LoD values had been established by the sponsor with the same strains on the investigational system: tenfold serial dilutions were tested in triplicate, followed by confirmatory testing of 20 replicates at concentrations near the estimated LoD, and the LoD was defined as the lowest concentration giving at least 95% positive results, a result being positive when the predefined target-specific cycle threshold and TraceR acceptance criteria were met. The LoD was not re-established in this study. The matrix was a pool of negative clinical nasopharyngeal specimens confirmed negative with FilmArray RP2.1 before spiking. For each target and concentration, a common spiked stock was prepared and divided into separate aliquots, each of which was processed and tested individually.

### 2.7. Operator Questionnaire

After completion of testing, the operators completed a post-study questionnaire, specified in the protocol before testing began, assessing the ease of instrument setup and operation, comprehensibility of the labelling, adherence to the testing instructions, clarity of the on-screen prompts, and any difficulties encountered in the workflow. The protocol version of the questionnaire rated each item on a 10-point scale from 1 (very easy) to 10 (very hard) and recorded each operator’s age, occupation, education and years of laboratory experience; the clinical study report summarized the responses only as “easy” or “very easy” and did not report item-level scores or operator characteristics. The questionnaire was not a validated usability instrument, and its results are reported as descriptive operator feedback rather than as a formal usability or human-factors evaluation. Ten operators performed investigational testing: four at the sponsor’s laboratory and three at each of the other two investigational testing laboratories. Together they performed 269 instrument runs, excluding quality-control-only runs; individual run counts were available only for the four operators whose runs included invalid results (174 runs; Table S9), and the other six operators performed the remaining 95 runs. No adverse events attributable to specimen collection or to the performance of either the investigational system or the comparator method were reported during the study.

### 2.8. Statistical Analysis

PPA was calculated as TP/(TP + FN) and NPA as TN/(TN + FP), where a true positive denotes agreement between the investigational system and the comparator on a positive result for a given target, and a true negative denotes agreement on a negative result. A false negative is an investigational negative with a positive comparator result, and a false positive is the converse. These terms describe disagreement relative to the comparator and do not imply that the investigational result was in error; in the text, discordances are described by direction (investigational-negative/comparator-positive or investigational-positive/comparator-negative). Agreement was computed per target such that each specimen contributed to the negative denominator of every target it did not carry. Two-sided 95% confidence intervals were obtained using the Wilson score method, which behaves acceptably at proportions close to unity and with small denominators [21,22]; agreement measures follow the definitions in CLSI EP12 [23]. Targets with no positive specimens in a given set were reported as unevaluable. A target met the prospective acceptance criteria only if both the PPA point estimate and the lower 95% confidence bound met the prespecified values; targets were classified as meeting all criteria, not meeting the lower-bound criterion, or not evaluable. Results from the prospective, archived and contrived sets were analyzed and reported separately and were not pooled. Invalid results were resolved by repeated testing of the original specimen. An initial invalid result was an invalid result on the first test of a specimen; a final invalid result was a specimen that remained invalid after repeat testing. Specimens that remained invalid by either method were excluded from the agreement analysis and reported separately. Detection rates were descriptive and not statistically compared between sites or age strata. Agreement estimates and Wilson score intervals were recalculated by the academic authors from the per-target counts in the clinical study report with a Python script (Python 3.13; Python Software Foundation, Wilmington, DE, USA; Supplementary File S1).

### 2.9. Sample Size

The protocol specified a target enrolment of approximately 1500 participants. The sample size was calculated with the method of Buderer for diagnostic accuracy studies [24], assuming a sensitivity of 90%, a specificity of 95%, a margin of error of 5% and an average target prevalence of 10%; the protocol reports a result of 1463 participants. On recalculation with these assumptions and a two-sided 95% confidence level, the sensitivity requirement gives 1383 participants and the specificity requirement 82. The protocol value of 1463 could not be reproduced from these documented assumptions, and the protocol does not state how it was derived. The planned enrolment of approximately 1500 and the 1482 evaluable prospective specimens exceeded both recalculated requirements. Consented enrolment was planned until 31 January 2026 or until 1000 specimens had been collected, after which residual specimens were added to reach the target. Because the calculation used an average prevalence across targets, it did not ensure that each target would accrue enough prospective positives to be evaluated against the acceptance criteria with adequate precision; the protocol therefore prespecified supplementation with archived and contrived specimens for low-prevalence targets (Section 2.6).

## 3. Results

### 3.1. Specimen Disposition and Study Population

A total of 1501 patients were prospectively enrolled in this study. Of them, 5 were excluded from the study; four did not meet the eligibility criteria, and one voluntarily withdrew from the study, leaving 1496 specimens from 1496 participants. Additionally, 11 specimens were rejected at receipt: seven due to transport temperature excursion, and four due to insufficient volume. Therefore, 1485 specimens were ultimately evaluated in this study. Of these, 94.1% (1397/1485) were fresh and 5.9% (88/1485) were frozen. Three specimens failed to yield valid testing results, leaving 1482 specimens for agreement analysis (Figure 1). Enrolment was distributed almost evenly across the three sites (Table 2).

The prospective population spanned the full intended use age range and was weighted towards children by the inclusion of a pediatric primary care site; the youngest stratum of patients was aged 2–6 years, while the oldest stratum contributed almost entirely to the hospital-based site. Participants younger than 18 years accounted for 54.0% (802/1485) of the accepted prospective cohort, and 11.0% (163/1485) were older than 65 years. Female participants accounted for 54.9% of the entire cohort. Moreover, 25.0% of the participants were of Hispanic ethnicity, with substantial variation between sites (Table 2). Among the 240 archived specimens, 55.4% (133/240) were from children younger than seven years, with a balanced sex distribution.

### 3.2. Validity of Results

On first testing, 10 of 1485 prospective specimens (0.67%) gave an initial invalid result on the investigational system and 11 (0.74%) on the comparator. Each was retested once, requiring 10 and 11 additional tests (in 7 and 11 instrument runs, respectively), and 9 specimens in each group gave valid results on retesting (Table S9). No run-level failure, defined as an instrument or run fault invalidating more than one specimen, occurred with either method; where a run contained more than one invalid specimen, the other specimens in that run were unaffected. Of the 269 investigational instrument runs, excluding quality-control-only runs, 9 (3.35%) contained at least one invalid specimen result. After repeat testing, the investigational system had a final invalid rate of 0.07% (1/1485), whereas the comparator had a final invalid rate of 0.13% (2/1485), in both cases because the repeat test also failed. All archived (240/240) and contrived (200/200) specimens yielded valid results.

### 3.3. Agreement in Prospective Specimens

Overall, 19 of 22 targets were detected at least once in prospective specimens (Table 3). Against the prespecified criteria, the 22 targets fell into three groups (Section 3.9). Twelve targets met all prospective acceptance criteria: adenovirus, HCoV-229E, HCoV-HKU1, HCoV-NL63, HCoV-OC43, SARS-CoV-2, human metapneumovirus, human rhinovirus/enterovirus, influenza A virus, influenza A virus H3, influenza B virus and RSV. Their PPA ranged from 91.5% to 100%, with lower 95% confidence bounds of at least 82.3%; for SARS-CoV-2, PPA was 97.0% (65/67; 95% CI 89.8–99.2), meeting the higher threshold of 95%. The negative percent agreement (NPA) ranged from 99.6% to 100%, with a lower confidence bound of at least 99.1% for every target, meeting the prespecified NPA criterion for all 22 targets.

Seven targets had PPA point estimates of at least 90% but lower 95% confidence bounds below 80.0% and therefore did not meet the complete prospective criterion: influenza A virus H1-2009 (100%, 13/13; lower bound 77.2%), parainfluenza virus 1 (90.0%, 9/10; 59.6%), parainfluenza virus 2 (100%, 5/5; 56.6%), parainfluenza virus 3 (100%, 7/7; 64.6%), parainfluenza virus 4 (100%, 8/8; 67.6%), *C. pneumoniae* (100%, 1/1; 20.7%) and *M. pneumoniae* (94.4%, 17/18; 74.2%). These targets did not meet the acceptance criterion; because the estimates rest on 1 to 18 positives, they are also too imprecise to characterize agreement for these targets. Three additional targets, influenza A virus H1, *B. parapertussis* and *B. pertussis*, had no positive prospective specimen, so PPA could not be estimated. In accordance with the protocol, these 10 targets were additionally tested in archived and/or contrived specimens (Section 3.7 and Section 3.8); those results are reported separately and do not change the prospective classification.

Human rhinovirus/enterovirus produced the largest number of positives and the lowest PPA point estimate among the well-represented targets (91.5%, 172/188; 95% CI 86.6–94.7), reflecting 16 specimens that were positive by the comparator and negative by the investigational system. Agreement was broadly consistent between fresh and frozen specimens, although several targets had only single-digit numbers of frozen positives. The small number of frozen positives per target implies that these subgroup estimates are imprecise; they have been reported in Table S1.

### 3.4. Adjudication of Discordant Results

Specimens with a discordant result for any target in the prospective or archived set were retested with the third assay, giving 86 target-level discordances (68 prospective and 18 archived): 45 investigational-negative/comparator-positive and 41 investigational-positive/comparator-negative. The third-assay result matched the comparator in 31 (36.0%) and the investigational system in 55 (64.0%) (Table S6). Because the third assay was applied only to discordant specimens and is itself a molecular panel with its own target design and analytical sensitivity, these results do not establish which result was correct, and they were not used to reclassify any result.

Of the 35 prospective investigational-negative/comparator-positive discordances, the third assay detected the target in 9 and did not detect it in 26. Of the 33 prospective investigational-positive/comparator-negative discordances, it detected the target in 16 and did not detect it in 17. Human rhinovirus/enterovirus accounted for 21 of the 68 prospective discordances (16 and 5 in the two directions), and the third assay matched the comparator in 3 of 16 and 3 of 5, respectively. Possible explanations for discordance include differences in primer and probe design, differences in analytical sensitivity, stochastic detection at concentrations near the LoD of one or more assays, and storage of the aliquot before retesting; because cycle threshold values were not analyzed, these explanations could not be distinguished. Discordances are listed by target, specimen set and direction in Table S6.

### 3.5. Detection Rates by Site and Age

At least one target was detected in 870 of 1482 evaluable specimens, with 1124 target-level detections; because influenza A subtype calls are counted in addition to the influenza A result, these correspond to 995 distinct organism detections. Human rhinovirus/enterovirus was the most frequently detected target (11.9%, 177/1482), followed by RSV (10.7%) and influenza A virus (8.7%). Bacterial targets accounted for 21 of the 995 organism detections (2.1%): 20 *M. pneumoniae* and a single *C. pneumoniae*, with no *Bordetella* species detected. Detection rates differed markedly between sites: influenza B virus was detected in 13.5% and 1.9% of specimens at Site 1 and Site 2, respectively, SARS-CoV-2 was detected in 8.4% of specimens at Site 3 and in no specimen at Site 2, whereas *M. pneumoniae* was detected in 3.9% of specimens at Site 2 compared with 0.2% and 0% at the other two sites (Table S3). These site differences were not adjusted for age or other factors and are reported descriptively.

Age stratification, in which the youngest stratum spanned 2–6 years because enrolment required an age of at least 2 years, showed the following patterns (Figure 2). RSV was detected in 23.8% of specimens from children aged 2–6 years, but only 4.9% of specimens from adults older than 65 years. Similarly, human rhinovirus/enterovirus was detected in 20.0% of specimens from the youngest stratum and only 6.1% of specimens from the oldest stratum. SARS-CoV-2 showed an inverse gradient, increasing from 4.1% in the youngest stratum to 11.0% in the oldest stratum. *M. pneumoniae* and influenza B viruses were most frequent in school-aged children.

### 3.6. Co-Detections

The investigational system reported more than one organism in 108 prospective specimens, excluding influenza A subtyping combinations, which represents 7.3% (108/1482) of the evaluable specimens. Among the 870 specimens positive for at least one target, 12.4% (108/870) contained multiple organisms. Most specimens contained two organisms (87.0%, 94/108), 11 specimens (10.2%) had three organisms, and three specimens (2.8%) had four organisms. In 15 of these 108 specimens (13.9%), at least one of the reported organisms was not detected by the comparator (Table S4). In the other direction, the investigational system did not detect at least one comparator-reported organism in 15 specimens in which the comparator reported more than one organism (12 with two organisms, two with three and one with four; Table S5). These 15 specimens carried 12 distinct comparator combinations, which occurred in 42 specimens in total; the investigational system reproduced the comparator result in full in the other 27 of these 42 specimens. The two groups of 15 specimens refer to opposite directions of disagreement. Detection of more than one organism by a molecular assay does not by itself establish co-infection.

### 3.7. Agreement in Archived Specimens

All 240 archived specimens yielded valid results using both methods. The archived set contributed a substantial number of positives for parainfluenza viruses (n = 36, 31, 30, and 29 for types 1 to 4, respectively), influenza A virus H1-2009 (n = 32), *M. pneumoniae* (n = 28), adenovirus (n = 27), and human rhinovirus/enterovirus (n = 25); specifically, the targets that the prospective collection had left imprecise. Because these specimens were selected on their historical result, their PPA values describe agreement in enriched material and are not estimates for a clinical population. The PPA was 100% for 12 of the 17 evaluable targets, and the NPA was at least 99.1% for every target (Table 4).

Archived PPA was lower than prospective PPA for four targets. The PPA for adenovirus was 88.9% (24/27), whereas the prospective PPA was 94.6%. The PPA for human metapneumovirus was 50.0% (2/4), although this estimate included only four positives, and the corresponding prospective estimate was 100% (79/79). The PPA for human rhinovirus/enterovirus was 88.0% (22/25) on archived material, compared with 91.5% (172/188) prospectively, and the PPA for parainfluenza virus 4 was 96.6% (28/29), compared with 100% (8/8) prospectively. In the three adenovirus and two human metapneumovirus discordances, the third assay did not detect the target (Table S6).

### 3.8. Agreement in Contrived Specimens

All 200 contrived specimens yielded valid results. For each of the four targets that the prospective and archived collections failed to represent, all contrived positive specimens were detected at each concentration tested (2×, 5× and 100× LoD; 50/50 per target), and no target was reported in contrived specimens that did not contain it (NPA 100%; Table 5). Because the specimens at each concentration were aliquots of a common spiked stock, these results are replicate tests of one preparation per concentration, and their confidence intervals reflect replicate testing rather than independent specimens. They describe detection of the specified strains at the specified concentrations in negative clinical matrix (Table S8) and are not estimates of clinical sensitivity in naturally infected patients.

### 3.9. Summary of Evidence by Target

Table 6 and Figure 3 summarize, for each target, the classification against the prospective acceptance criteria and the positives contributed by each specimen set. Results from the three sets were not pooled, because the sets differ in sampling mechanism and, for contrived specimens, in the reference used (known composition rather than the comparator). For influenza A virus H1 and *B. parapertussis*, positive evidence came only from contrived specimens; for *B. pertussis*, from one archived and 50 contrived specimens; and for *C. pneumoniae*, from one prospective, four archived and 50 contrived specimens. For the other six targets that did not meet the prospective criterion (influenza A virus H1-2009, parainfluenza viruses 1 to 4 and *M. pneumoniae*), archived specimens provided 28 to 36 additional positives per target, with PPA of 96.4% to 100%.

### 3.10. Operator Feedback

Overall, nine of the ten operators completed a post-study questionnaire; one operator did not complete the questionnaire before the study concluded. All instrument setups and routine operations were rated as easy or very easy, and none of the operators reported instrument problems or specific difficulties with the testing procedure. Review of the 11 invalid investigational test results (10 initial tests and 1 repeat test) did not identify a documented operator error contributing to them. These are descriptive responses from trained operators and do not constitute a formal usability evaluation.

## 4. Discussion

In this prospective multicenter study spanning one respiratory season, the investigational system showed high agreement with the comparator for the 12 targets that were well represented in prospective specimens, meeting all prespecified criteria, and NPA was at least 99.6% for all 22 targets. For seven further targets, the prospective PPA point estimates were at least 90%, but the lower confidence bounds were below 80% because positives were few, and three targets had no prospective positives. For these ten targets, the prospective data do not establish that the acceptance criteria were met. The study was not designed as an equivalence or non-inferiority comparison, and its results describe agreement between two molecular panels rather than accuracy against a reference standard. For the well-represented targets, the observed agreement is within the range reported in evaluations of the comparator platform and other syndromic panels [6,7,8,9,10,11], although differences in design, comparator and population limit direct comparison.

Two features of the results require comment: invalid results and human rhinovirus/enterovirus. First, initial invalid results occurred in 0.67% of specimens on the investigational system and 0.74% on the comparator; each required one additional test, no run-level failure occurred, and the final invalid rates after repeat testing were 0.07% and 0.13%, respectively. Because published evaluations of cartridge-based panels report failed runs and repeat testing with different definitions [6,9,11], we do not compare these rates across studies. Hands-on time, turnaround time and throughput were not measured, so the effect of repeat testing on laboratory workflow could not be assessed.

Second, human rhinovirus/enterovirus had the lowest prospective PPA among the well-represented targets (91.5%, with 16 investigational-negative/comparator-positive specimens) and was the most frequently detected target. The rhinovirus species comprise more than 160 genotypes with extensive sequence divergence in the regions typically targeted. Additionally, the shared 5′ untranslated region makes their discrimination from the enteroviruses difficult; discordance between assays for this target is a recurring finding rather than a peculiarity of this study [25,26]. The third assay detected the target in 3 of these 16 specimens. Because it was applied only to discordant specimens and has its own target design and analytical sensitivity, this does not show which assay was correct. Differences in target regions, analytical sensitivity near the LoD and storage of the aliquot before retesting are possible explanations that this study could not separate without cycle threshold values. The prospective PPA therefore remains the primary estimate. Nonetheless, laboratories should not treat a negative rhinovirus/enterovirus result from any current panel as excluding infection.

The supplementation strategy implemented in this study merits attention because it is both a strength and a constraint on interpretation. In total, seven targets had 1–18 comparator-positive prospective specimens, and three additional targets were not detected; thus, 10 targets had fewer than 20 positive prospective specimens, which is an unavoidable consequence of evaluating a broad panel over one season in one hemisphere. Prospective collection alone gave wide confidence intervals for influenza A virus H1-2009, the parainfluenza viruses, *M. pneumoniae* and *C. pneumoniae*, and no estimate for influenza A virus H1 and both *Bordetella* species; supplementation with banked and contrived material is the approach anticipated by regulatory guidance for such targets [18]. The archived specimens provide supplementary agreement data in naturally infected specimens for six of these targets, but they were selected on historical result, are not population estimates and do not change the prospective classification. The contrived specimens provide analytical evidence of detection of the specified strains at 2×, 5× and 100× LoD in clinical matrix, but they cannot represent the sequence diversity of circulating strains, the range of concentrations or inhibitory substances present in real specimens. Clinical performance therefore remains unestablished for influenza A virus H1 and *B. parapertussis* (contrived specimens only) and is supported by very limited clinical material for *B. pertussis* (one archived positive) and *C. pneumoniae* (one prospective and four archived positives). Evaluation in seasons or settings in which these organisms circulate is needed.

The proportion of specimens with more than one organism detected (7.3% of evaluable and 12.4% of positive specimens) is consistent with those obtained in a contemporary syndromic panel series and with reports of concurrent detection of SARS-CoV-2 and other respiratory pathogens [27]. Molecular detection of more than one organism does not establish co-infection, and molecular detection alone is not evidence of causation, particularly for rhinovirus and adenovirus, which are recovered from a substantial proportion of asymptomatic children [28].

The age-stratified detection rates (Figure 2) showed RSV and rhinovirus/enterovirus most often in children aged 2–6 years, influenza B and *M. pneumoniae* in school-age children, and SARS-CoV-2 most often in adults older than 65 years. These are descriptive observations from three sites in one season, not adjusted for site or other factors, and they do not provide evidence on assay specificity.

Clinical management was outside the scope of this study. Of the 995 distinct organism detections in the prospective set, 21 (2.1%) were bacterial (20 *M. pneumoniae* and one *C. pneumoniae*), and no *Bordetella* species was detected. A viral detection does not establish that the virus is causing the current illness, does not exclude bacterial co-infection, and the panel has no target for typical respiratory bacterial pathogens such as *Streptococcus pneumoniae* or *Haemophilus influenzae* [18]. The panel does not detect macrolide resistance in *M. pneumoniae*, which is common in parts of Asia [29,30]. Randomized evidence indicates that making a rapid respiratory result available does not by itself reduce antibiotic prescribing [12,13]. This study did not collect final diagnoses, disease severity, antibiotic prescribing, patient outcomes, turnaround time or cost, so it provides no evidence on whether testing with this system changes clinical management or antibiotic use; this would need to be assessed in dedicated studies.

### Limitations

This study has several limitations. First, the comparator was a single molecular panel rather than a composite reference standard or culture with sequencing; therefore, agreement statistics describe the concordance between two amplification assays and cannot separate the sensitivity of one from that of the other. The third assay was applied only to discordant specimens, so it provides no reference result for concordant specimens and cannot assign error to either method. Second, the study was not designed or powered as an equivalence or non-inferiority study, and the sample-size calculation used an average prevalence across targets, so the total enrolment did not provide enough prospective positives to evaluate ten targets against the acceptance criteria. Third, for influenza A virus H1 and *B. parapertussis*, the only positive evidence came from contrived specimens, and for *B. pertussis* and *C. pneumoniae*, clinical positives were very few; archived specimens were selected on historical result, contrived specimens cannot represent natural strain diversity, pathogen loads or matrix effects, and the contrived specimens at each concentration were aliquots of a single spiked stock, so their results reflect replicate testing rather than independent specimens. Fourth, the manufacturer funded the study, designed the protocol, reviewed the manuscript and operated a laboratory that served as an investigational testing laboratory for one site and as the comparator laboratory for the other two, and the CRO that managed the study shares ownership with one investigational testing laboratory. Although no specimen was tested by both methods in the same laboratory and the measures described in Section 2.5 were used to keep the methods blinded, an independent replication would strengthen the evidence; in addition, the academic authors verified the analysis only from the per-target counts in the clinical study report; they did not re-derive these counts from the line-level dataset, which the sponsor has since authorized the corresponding authors to provide, and did not examine instrument output. Fifth, enrolment covered a single Northern Hemisphere season at three US sites; therefore, the observed strain complement and the relative prevalence cannot be generalized to other regions or seasons. Sixth, final clinical diagnoses, disease severity, radiographic findings, antibiotic prescribing, patient outcomes, turnaround time, hands-on time, throughput and cost were not collected, so no conclusion about clinical utility or operational benefit can be drawn. Seventh, only nasopharyngeal swabs in the universal transport medium were evaluated, and cycle threshold values were not analyzed, so discordances could not be related to target concentration. Finally, the operator questionnaire was not a validated instrument and was completed after training and testing, so it provides descriptive feedback only.

## 5. Conclusions

In this three-site prospective study, a fully integrated sample-to-answer multiplex PCR respiratory panel showed high agreement with the comparator syndromic panel for the 12 targets that were well represented in prospective specimens, with NPA of at least 99.6% for all 22 targets and a final invalid rate of 0.07% after repeat testing. For the remaining ten targets, the prospective data were insufficient to confirm the prespecified acceptance criteria; archived specimens provide supplementary agreement data in enriched clinical material and contrived specimens provide analytical evidence of detection, but neither changes the prospective classification of these targets. Future studies in other seasons and regions, in independent laboratories, and with turnaround time, workflow and clinical outcome endpoints are required.

## Figures and Tables

**Figure 2:**
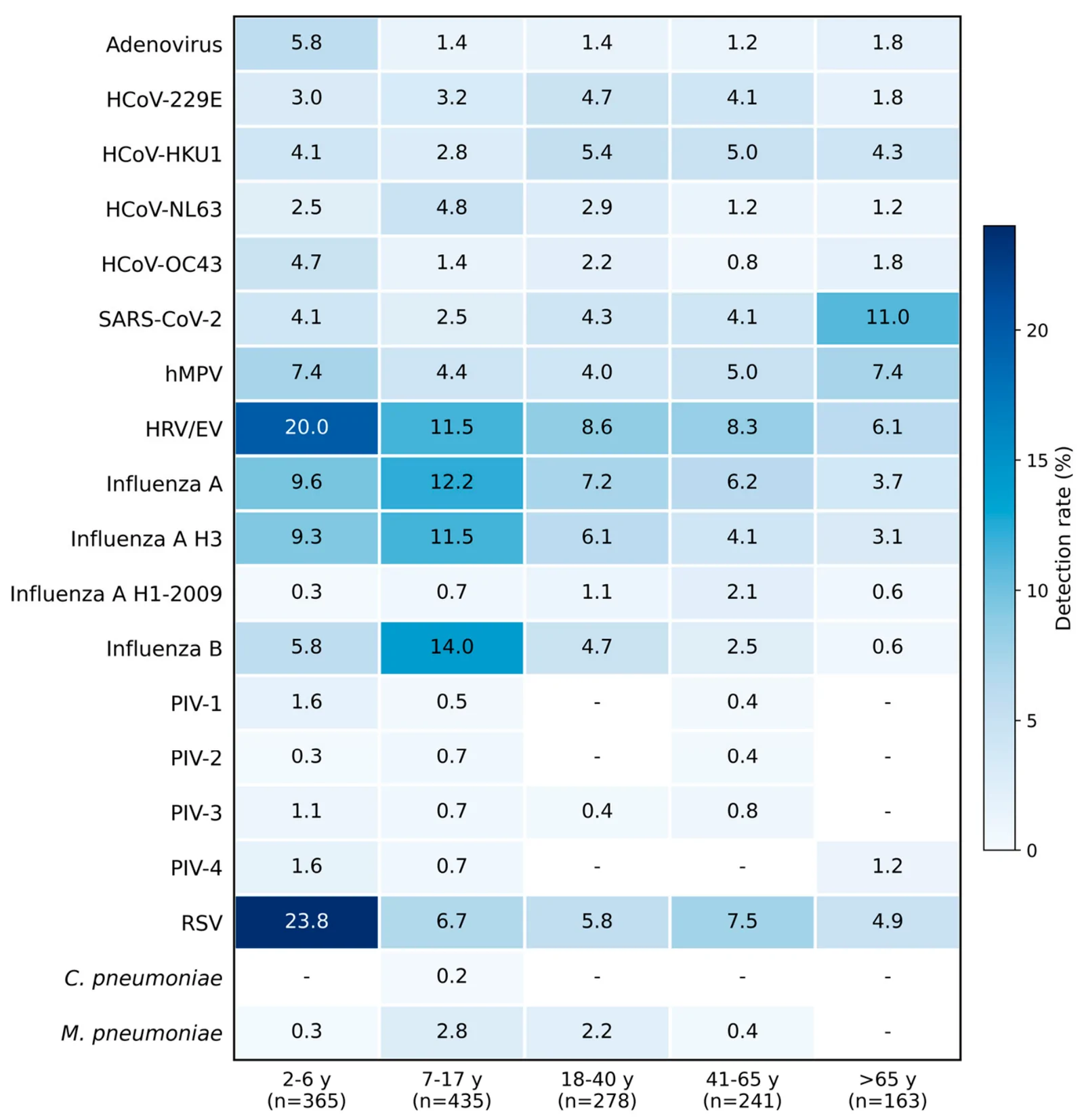
Detection rate of each target by the investigational system in the 1482 evaluable prospective specimens, stratified by age group. The denominator of every cell is all prospective specimens in that age stratum, and not the specimens positive for any target. This ensures that the values obtained are detection rates in the tested population rather than a distribution among positives. Stratum sizes are given beneath each column label. The youngest stratum begins at 2 years because that was the lower age limit for enrolment. A dash indicates no detection; targets with no detection in any stratum were omitted. These are descriptive epidemiological observations from a single season at three sites and are secondary to the agreement results in Table 3. They are not adjusted for site or other factors and do not provide evidence on assay specificity.

**Figure 3:**
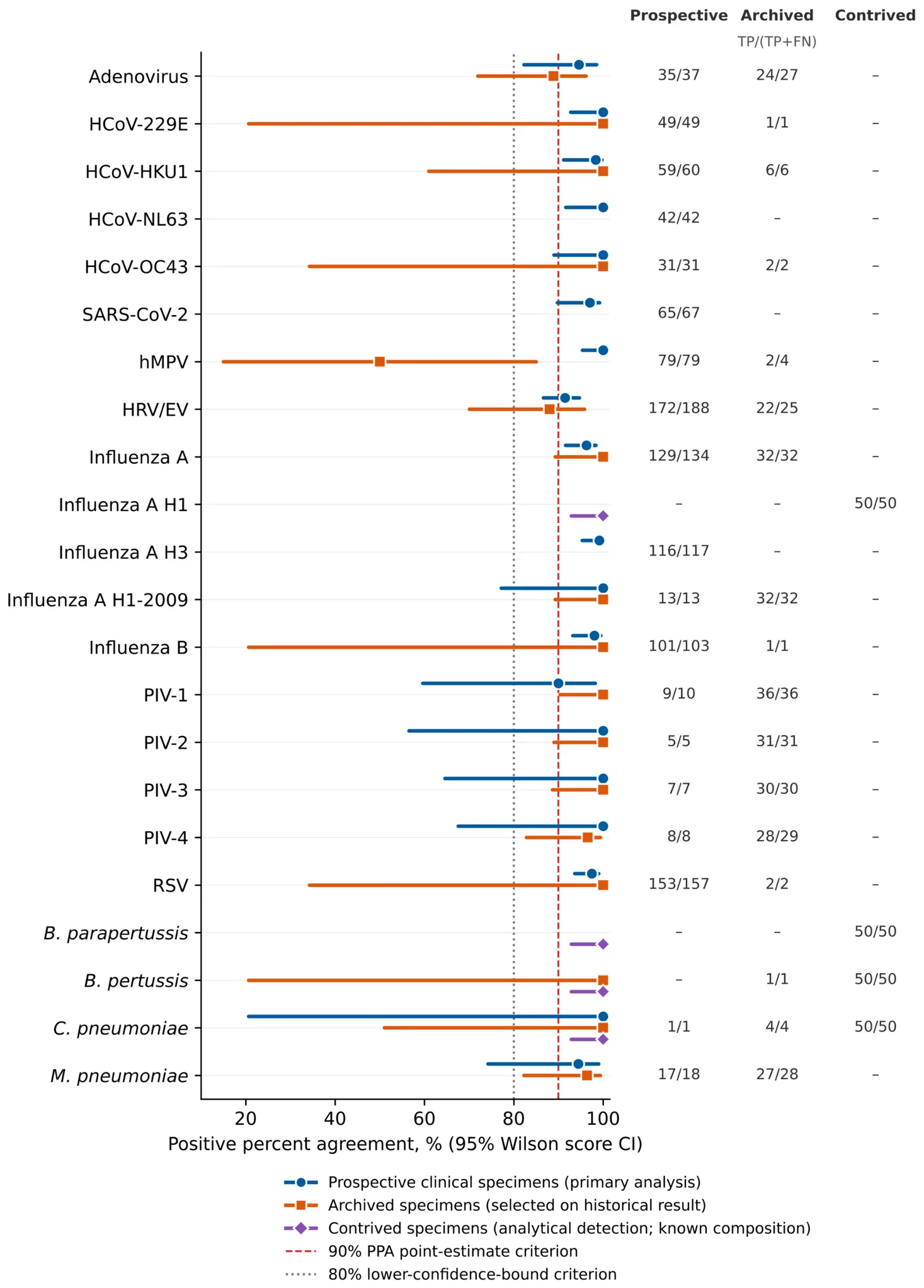
Positive percent agreement by target, shown separately for each specimen set. Circles indicate prospective clinical specimens, which constitute the primary analysis; squares indicate archived specimens, selected on historical result and enriched for their targets; diamonds indicate contrived specimens, prepared at fixed multiples of the limit of detection, for which the known composition was the reference. Results were not pooled across sets. For contrived specimens, the intervals reflect replicate testing of a single spiked preparation per concentration. Horizontal bars indicate the Wilson score 95% confidence intervals. The pre-specified acceptance criterion required a point estimate of at least 90% (dashed line) and a lower 95% confidence bound of at least 80.0% (dotted line); for SARS-CoV-2 the required point estimate was 95% rather than 90%, and that target is not marked separately on the figure. Counts to the right are TP/(TP + FN) for each set; a dash indicates that the target had no positive specimen in that set.

**Table 2:** Demographic characteristics of the 1485 prospectively collected specimens accepted for testing.

Characteristic		Overall (n = 1485)	Site 1 (n = 499)	Site 2 (n = 486)	Site 3 (n = 500)
Age group	2–6 years	366 (24.6)	194 (38.9)	57 (11.7)	115 (23.0)
	7–17 years	436 (29.4)	238 (47.7)	153 (31.5)	45 (9.0)
	18–40 years	278 (18.7)	39 (7.8)	138 (28.4)	101 (20.2)
	41–65 years	242 (16.3)	28 (5.6)	121 (24.9)	93 (18.6)
	>65 years	163 (11.0)	0 (0.0)	17 (3.5)	146 (29.2)
Sex	Male	667 (44.9)	233 (46.7)	195 (40.1)	239 (47.8)
	Female	815 (54.9)	265 (53.1)	291 (59.9)	259 (51.8)
	Other/not reported	3 (0.2)	1 (0.2)	0 (0.0)	2 (0.4)
Ethnicity	Hispanic	371 (25.0)	123 (24.6)	219 (45.1)	29 (5.8)
	Non-Hispanic	1114 (75.0)	376 (75.4)	267 (54.9)	471 (94.2)
Race	White	961 (64.7)	154 (30.9)	420 (86.4)	387 (77.4)
	Asian	160 (10.8)	98 (19.6)	44 (9.1)	18 (3.6)
	Black or African American	102 (6.9)	47 (9.4)	6 (1.2)	49 (9.8)
	American Indian or Alaska Native	1 (0.1)	0 (0.0)	1 (0.2)	0 (0.0)
	Native Hawaiian or Other Pacific Islander	1 (0.1)	0 (0.0)	1 (0.2)	0 (0.0)
	Other	260 (17.5)	200 (40.1)	14 (2.9)	46 (9.2)

Values are n (%) of the column total.

**Table 3:** Agreement of the investigational system with the comparator method in 1482 evaluable prospective nasopharyngeal specimens.

Target	TP/(TP + FN)	PPA, % (95% CI)	TN/(TN + FP)	NPA, % (95% CI)
Adenovirus	35/37	94.6 (82.3–98.5)	1443/1445	99.9 (99.5–100.0)
HCoV-229E	49/49	100.0 (92.7–100.0)	1431/1433	99.9 (99.5–100.0)
HCoV-HKU1	59/60	98.3 (91.1–99.7)	1420/1422	99.9 (99.5–100.0)
HCoV-NL63	42/42	100.0 (91.6–100.0)	1439/1440	99.9 (99.6–100.0)
HCoV-OC43	31/31	100.0 (89.0–100.0)	1448/1451	99.8 (99.4–99.9)
SARS-CoV-2	65/67	97.0 (89.8–99.2)	1414/1415	99.9 (99.6–100.0)
Human metapneumovirus	79/79	100.0 (95.4–100.0)	1401/1403	99.9 (99.5–100.0)
Human rhinovirus/enterovirus	172/188	91.5 (86.6–94.7)	1289/1294	99.6 (99.1–99.8)
Influenza A virus	129/134	96.3 (91.6–98.4)	1348/1348	100.0 (99.7–100.0)
Influenza A virus H1	0/0	NE	1482/1482	100.0 (99.7–100.0)
Influenza A virus H3	116/117	99.1 (95.3–99.8)	1365/1365	100.0 (99.7–100.0)
Influenza A virus H1-2009	13/13	100.0 (77.2–100.0)	1469/1469	100.0 (99.7–100.0)
Influenza B virus	101/103	98.1 (93.2–99.5)	1378/1379	99.9 (99.6–100.0)
Parainfluenza virus 1	9/10	90.0 (59.6–98.2)	1472/1472	100.0 (99.7–100.0)
Parainfluenza virus 2	5/5	100.0 (56.6–100.0)	1477/1477	100.0 (99.7–100.0)
Parainfluenza virus 3	7/7	100.0 (64.6–100.0)	1472/1475	99.8 (99.4–99.9)
Parainfluenza virus 4	8/8	100.0 (67.6–100.0)	1471/1474	99.8 (99.4–99.9)
Respiratory syncytial virus	153/157	97.5 (93.6–99.0)	1320/1325	99.6 (99.1–99.8)
*Bordetella parapertussis*	0/0	NE	1482/1482	100.0 (99.7–100.0)
*Bordetella pertussis*	0/0	NE	1482/1482	100.0 (99.7–100.0)
*Chlamydia pneumoniae*	1/1	100.0 (20.7–100.0)	1481/1481	100.0 (99.7–100.0)
*Mycoplasma pneumoniae*	17/18	94.4 (74.2–99.0)	1461/1464	99.8 (99.4–99.9)

PPA, positive percent agreement; NPA, negative percent agreement; TP, true positive; FN, false negative; TN, true negative; FP, false positive; NE, not evaluable. TP, FN, TN and FP are defined relative to the comparator and do not imply that either method was correct. The confidence intervals are Wilson score intervals. The results for fresh and frozen specimens are listed in Table S1.

**Table 4:** Agreement in 240 archived nasopharyngeal specimens.

Target	TP/(TP + FN)	PPA, % (95% CI)	TN/(TN + FP)	NPA, % (95% CI)
Adenovirus	24/27	88.9 (71.9–96.1)	212/213	99.5 (97.4–99.9)
HCoV-229E	1/1	100.0 (20.7–100.0)	239/239	100.0 (98.4–100.0)
HCoV-HKU1	6/6	100.0 (61.0–100.0)	234/234	100.0 (98.4–100.0)
HCoV-NL63	0/0	NE	240/240	100.0 (98.4–100.0)
HCoV-OC43	2/2	100.0 (34.2–100.0)	238/238	100.0 (98.4–100.0)
SARS-CoV-2	0/0	NE	239/240	99.6 (97.7–99.9)
Human metapneumovirus	2/4	50.0 (15.0–85.0)	236/236	100.0 (98.4–100.0)
Human rhinovirus/enterovirus	22/25	88.0 (70.0–95.8)	214/215	99.5 (97.4–99.9)
Influenza A virus	32/32	100.0 (89.3–100.0)	208/208	100.0 (98.2–100.0)
Influenza A virus H1	0/0	NE	240/240	100.0 (98.4–100.0)
Influenza A virus H3	0/0	NE	240/240	100.0 (98.4–100.0)
Influenza A virus H1-2009	32/32	100.0 (89.3–100.0)	208/208	100.0 (98.2–100.0)
Influenza B virus	1/1	100.0 (20.7–100.0)	239/239	100.0 (98.4–100.0)
Parainfluenza virus 1	36/36	100.0 (90.4–100.0)	204/204	100.0 (98.2–100.0)
Parainfluenza virus 2	31/31	100.0 (89.0–100.0)	209/209	100.0 (98.2–100.0)
Parainfluenza virus 3	30/30	100.0 (88.6–100.0)	209/210	99.5 (97.4–99.9)
Parainfluenza virus 4	28/29	96.6 (82.8–99.4)	210/211	99.5 (97.4–99.9)
Respiratory syncytial virus	2/2	100.0 (34.2–100.0)	238/238	100.0 (98.4–100.0)
*Bordetella parapertussis*	0/0	NE	240/240	100.0 (98.4–100.0)
*Bordetella pertussis*	1/1	100.0 (20.7–100.0)	238/239	99.6 (97.7–99.9)
*Chlamydia pneumoniae*	4/4	100.0 (51.0–100.0)	236/236	100.0 (98.4–100.0)
*Mycoplasma pneumoniae*	27/28	96.4 (82.3–99.4)	210/212	99.1 (96.6–99.7)

Twenty known negative specimens were included in the set for blinding and randomization. PPA, positive percent agreement; NPA, negative percent agreement; TP, FN, TN and FP are defined relative to the comparator; NE, not evaluable. Archived specimens were selected based on historical results, and these estimates are not clinical population estimates.

**Table 5:** Agreement in 200 contrived specimens prepared for four low-prevalence targets.

Target	Concentration	TP/(TP + FN)	PPA, % (95% CI)	TN/(TN + FP)	NPA, % (95% CI)
Influenza A virus H1	2× LoD	30/30	100.0 (88.6–100.0)	90/90	100.0 (95.9–100.0)
	5× LoD	10/10	100.0 (72.2–100.0)	30/30	100.0 (88.6–100.0)
	100× LoD	10/10	100.0 (72.2–100.0)	30/30	100.0 (88.6–100.0)
	Combined	50/50	100.0 (92.9–100.0)	150/150	100.0 (97.5–100.0)
*Bordetella parapertussis*	2× LoD	30/30	100.0 (88.6–100.0)	90/90	100.0 (95.9–100.0)
	5× LoD	10/10	100.0 (72.2–100.0)	30/30	100.0 (88.6–100.0)
	100× LoD	10/10	100.0 (72.2–100.0)	30/30	100.0 (88.6–100.0)
	Combined	50/50	100.0 (92.9–100.0)	150/150	100.0 (97.5–100.0)
*Bordetella pertussis*	2× LoD	30/30	100.0 (88.6–100.0)	90/90	100.0 (95.9–100.0)
	5× LoD	10/10	100.0 (72.2–100.0)	30/30	100.0 (88.6–100.0)
	100× LoD	10/10	100.0 (72.2–100.0)	30/30	100.0 (88.6–100.0)
	Combined	50/50	100.0 (92.9–100.0)	150/150	100.0 (97.5–100.0)
*Chlamydia pneumoniae*	2× LoD	30/30	100.0 (88.6–100.0)	90/90	100.0 (95.9–100.0)
	5× LoD	10/10	100.0 (72.2–100.0)	30/30	100.0 (88.6–100.0)
	100× LoD	10/10	100.0 (72.2–100.0)	30/30	100.0 (88.6–100.0)
	Combined	50/50	100.0 (92.9–100.0)	150/150	100.0 (97.5–100.0)

LoD, limit of detection. Thirty specimens were prepared at 2× LoD and ten each at 5× and 100× LoD for each target. The known composition of each specimen was used as the reference. Specimens at each concentration were aliquots of a common spiked stock; strains and concentrations are listed in Table S8.

**Table 6:** Summary of evidence by target and specimen set.

Target	Prospective TP/(TP + FN)	Prospective PPA, % (95% CI)	Prospective Acceptance Criteria	Archived TP/(TP + FN)	Contrived Detected/Tested
Adenovirus	35/37	94.6 (82.3–98.5)	Met	24/27	–
HCoV-229E	49/49	100.0 (92.7–100.0)	Met	1/1	–
HCoV-HKU1	59/60	98.3 (91.1–99.7)	Met	6/6	–
HCoV-NL63	42/42	100.0 (91.6–100.0)	Met	0/0 (NE)	–
HCoV-OC43	31/31	100.0 (89.0–100.0)	Met	2/2	–
SARS-CoV-2	65/67	97.0 (89.8–99.2)	Met	0/0 (NE)	–
Human metapneumovirus	79/79	100.0 (95.4–100.0)	Met	2/4	–
Human rhinovirus/enterovirus	172/188	91.5 (86.6–94.7)	Met	22/25	–
Influenza A virus	129/134	96.3 (91.6–98.4)	Met	32/32	–
Influenza A virus H1	0/0	NE	NE (no positives)	0/0 (NE)	50/50
Influenza A virus H3	116/117	99.1 (95.3–99.8)	Met	0/0 (NE)	–
Influenza A virus H1-2009	13/13	100.0 (77.2–100.0)	Not met (lower bound < 80%)	32/32	–
Influenza B virus	101/103	98.1 (93.2–99.5)	Met	1/1	–
Parainfluenza virus 1	9/10	90.0 (59.6–98.2)	Not met (lower bound < 80%)	36/36	–
Parainfluenza virus 2	5/5	100.0 (56.6–100.0)	Not met (lower bound < 80%)	31/31	–
Parainfluenza virus 3	7/7	100.0 (64.6–100.0)	Not met (lower bound < 80%)	30/30	–
Parainfluenza virus 4	8/8	100.0 (67.6–100.0)	Not met (lower bound < 80%)	28/29	–
Respiratory syncytial virus	153/157	97.5 (93.6–99.0)	Met	2/2	–
*Bordetella parapertussis*	0/0	NE	NE (no positives)	0/0 (NE)	50/50
*Bordetella pertussis*	0/0	NE	NE (no positives)	1/1	50/50
*Chlamydia pneumoniae*	1/1	100.0 (20.7–100.0)	Not met (lower bound < 80%)	4/4	50/50
*Mycoplasma pneumoniae*	17/18	94.4 (74.2–99.0)	Not met (lower bound < 80%)	27/28	–

PPA, positive percent agreement; CI, confidence interval (Wilson score); NE, not evaluable (no positive specimen); –, not tested in that set. TP and FN are defined relative to the comparator for prospective and archived specimens and relative to known composition for contrived specimens. “Met” requires a PPA point estimate of at least 90% (95% for SARS-CoV-2) and a lower 95% confidence bound of at least 80.0%. NPA met the prespecified criterion for all 22 targets in prospective specimens (Table 3). Results from the three specimen sets were not pooled; archived and contrived results do not change the prospective classification.

## Data Availability

The per-target counts underlying all agreement estimates are reported in the article and Supplementary Materials, and the analysis code is provided as Supplementary File S1. The de-identified line-level dataset, the analysis code and the study protocol are available from the corresponding authors upon reasonable request for non-commercial research, subject to an executed data use agreement and applicable ethical and data-protection requirements. Under a standing written authorization from the sponsor dated 12 October 2026, the corresponding authors assess requests and provide these materials without case-by-case review or approval by the sponsor. Names of participating institutions, collection sites and testing laboratories are replaced with coded identifiers, because these organizations have not authorized the disclosure of their identities.

## Supplementary Materials

The following supporting information can be downloaded at: https://www.mdpi.com/article/10.3390/diagnostics16213541/s1, Table S1: Agreement stratified by fresh and frozen specimen categories; Table S2: demographic characteristics of the archived specimen set; Table S3: detection rate of each target by collection site; Table S4: multiple-organism combinations reported by the investigational system; Table S5: multiple-organism combinations reported by the comparator method in which the investigational system did not detect at least one organism; Table S6: results of discordance adjudication by target; Table S7: completed STARD 2015 checklist; Table S8: strains, limits of detection and concentrations of contrived specimens; Table S9: initial and final invalid results and repeat testing; File S1: Analysis code (Python) for the agreement estimates and confidence intervals.

## Abbreviations

The following abbreviations are used in this manuscript:

PPA	Positive percent agreement
NPA	Negative percent agreement
TP	True positive
FN	False negative
TN	True negative
FP	False positive
NE	Not evaluable
CI	Confidence interval
NPS	Nasopharyngeal swab
LoD	Limit of detection
EDC	Electronic data capture
CLIA	Clinical Laboratory Improvement Amendments
IRB	Institutional Review Board
RSV	Respiratory syncytial virus
hMPV	Human metapneumovirus
PIV	Parainfluenza virus
