# Supplementary material for: Clinical Performance of a Fully Integrated Sample-to-Answer Multiplex PCR Respiratory Panel: A Multicenter Evaluation Using Prospective, Archived and Contrived Nasopharyngeal Specimens
Source: Diagnostics (Basel). 2026 Oct 28;16(21):3541. doi: 10.3390/diagnostics16213541 (PMC13761482; doi:10.3390/diagnostics16213541)
Supplement: Supplementary file 1 [file diagnostics-16-03541-s001.zip › diagnostics-4604166-supplementary.pdf]

Supplementary Materials

Clinical Performance of a Fully Integrated Sample-to-Answer Multiplex PCR Respiratory Panel: A Multicenter Evaluation Using Prospective, Archived and Contrived Nasopharyngeal Specimens

All confidence intervals are Wilson score intervals. PPA, positive percent agreement; NPA, negative percent agreement; TP, true positive; FN, false negative; TN, true negative; FP, false positive; NE, not evaluable.

Table S1. Agreement in prospective specimens stratified by specimen category. Category I specimens were held at 2-8 °C and tested within 72 h; category II specimens were frozen below -20 °C.

Target	Fresh (category I): 1397 accepted, 1394 with paired valid results				Frozen (category II): 88 accepted, 88 with paired valid results			
	TP/(TP+FN)	PPA, % (95% CI)	TN/(TN+FP)	NPA, % (95% CI)	TP/(TP+FN)	PPA, % (95% CI)	TN/(TN+FP)	NPA, % (95% CI)
Adenovirus	30/32	93.8 (79.9–98.3)	1360/1362	99.9 (99.5–100.0)	5/5	100.0 (56.6–100.0)	83/83	100.0 (95.6–100.0)
HCoV-229E	44/44	100.0 (92.0–100.0)	1348/1350	99.9 (99.5–100.0)	5/5	100.0 (56.6–100.0)	83/83	100.0 (95.6–100.0)
HCoV-HKU1	57/57	100.0 (93.7–100.0)	1335/1337	99.9 (99.5–100.0)	2/3	66.7 (20.8–93.9)	85/85	100.0 (95.7–100.0)
HCoV-NL63	42/42	100.0 (91.6–100.0)	1351/1352	99.9 (99.6–100.0)	0/0	NE	88/88	100.0 (95.8–100.0)
HCoV-OC43	28/28	100.0 (87.9–100.0)	1365/1366	99.9 (99.6–100.0)	3/3	100.0 (43.9–100.0)	83/85	97.6 (91.8–99.4)
SARS-CoV-2	59/61	96.7 (88.8–99.1)	1332/1333	99.9 (99.6–100.0)	6/6	100.0 (61.0–100.0)	82/82	100.0 (95.5–100.0)
Human metapneumovirus	77/77	100.0 (95.2–100.0)	1315/1317	99.8 (99.4–100.0)	2/2	100.0 (34.2–100.0)	86/86	100.0 (95.7–100.0)
Human rhinovirus/enterovirus	165/179	92.2 (87.3–95.3)	1210/1215	99.6 (99.0–99.8)	7/9	77.8 (45.3–93.7)	79/79	100.0 (95.4–100.0)
Influenza A virus	126/131	96.2 (91.4–98.4)	1263/1263	100.0 (99.7–100.0)	3/3	100.0 (43.9–100.0)	85/85	100.0 (95.7–100.0)
Influenza A virus H1	0/0	NE	1394/1394	100.0 (99.7–100.0)	0/0	NE	88/88	100.0 (95.8–100.0)
Influenza A virus H3	114/115	99.1 (95.2–99.8)	1279/1279	100.0 (99.7–100.0)	2/2	100.0 (34.2–100.0)	86/86	100.0 (95.7–100.0)
Influenza A virus H1-2009	12/12	100.0 (75.8–100.0)	1382/1382	100.0 (99.7–100.0)	1/1	100.0 (20.7–100.0)	87/87	100.0 (95.8–100.0)
Influenza B virus	97/98	99.0 (94.4–99.8)	1295/1296	99.9 (99.6–100.0)	4/5	80.0 (37.6–96.4)	83/83	100.0 (95.6–100.0)
Parainfluenza virus 1	9/9	100.0 (70.1–100.0)	1385/1385	100.0 (99.7–100.0)	0/1	0.0 (0.0–79.3)	87/87	100.0 (95.8–100.0)
Parainfluenza virus 2	5/5	100.0 (56.6–100.0)	1389/1389	100.0 (99.7–100.0)	0/0	NE	88/88	100.0 (95.8–100.0)
Parainfluenza virus 3	7/7	100.0 (64.6–100.0)	1384/1387	99.8 (99.4–99.9)	0/0	NE	88/88	100.0 (95.8–100.0)
Parainfluenza virus 4	6/6	100.0 (61.0–100.0)	1385/1388	99.8 (99.4–99.9)	2/2	100.0 (34.2–100.0)	86/86	100.0 (95.7–100.0)
Respiratory syncytial virus	148/152	97.4 (93.4–99.0)	1238/1242	99.7 (99.2–99.9)	5/5	100.0 (56.6–100.0)	82/83	98.8 (93.5–99.8)
Bordetella parapertussis	0/0	NE	1394/1394	100.0 (99.7–100.0)	0/0	NE	88/88	100.0 (95.8–100.0)
Bordetella pertussis	0/0	NE	1394/1394	100.0 (99.7–100.0)	0/0	NE	88/88	100.0 (95.8–100.0)
Chlamydia pneumoniae	1/1	100.0 (20.7–100.0)	1393/1393	100.0 (99.7–100.0)	0/0	NE	88/88	100.0 (95.8–100.0)
Mycoplasma pneumoniae	17/18	94.4 (74.2–99.0)	1373/1376	99.8 (99.4–99.9)	0/0	NE	88/88	100.0 (95.8–100.0)

Fresh and frozen denominators sum to the overall denominators reported in Table 3 of the main text. Frozen-category estimates rest on few positives per target and are shown for completeness only.

Table S2. Demographic characteristics of the 240 archived nasopharyngeal specimens.

Characteristic	Category	n (%)
Age group	<7 years	133 (55.4)
	7-17 years	38 (15.8)
	18-40 years	23 (9.6)
	41-65 years	29 (12.1)
	>65 years	17 (7.1)
Sex	Male	118 (49.2)
	Female	122 (50.8)
Total		240 (100.0)

Ethnicity and race were not available for archived specimens, which were supplied de-identified. Twenty specimens known to be negative were included in the set for blinding and randomization.

Table S3. Detection rate of each target by the investigational system in 1482 evaluable prospective specimens, by collection site.

Target	Site 1, pediatric primary care, New Jersey (n = 498)	Site 2, urgent care, California (n = 486)	Site 3, hospital-based service, New York State (n = 498)	Overall (n = 1482)
Adenovirus	2.21 (11/498)	1.03 (5/486)	4.22 (21/498)	2.50 (37/1482)
HCoV-229E	5.02 (25/498)	1.85 (9/486)	3.41 (17/498)	3.44 (51/1482)
HCoV-HKU1	2.41 (12/498)	5.97 (29/486)	4.02 (20/498)	4.12 (61/1482)
HCoV-NL63	3.82 (19/498)	3.50 (17/486)	1.41 (7/498)	2.90 (43/1482)
HCoV-OC43	2.41 (12/498)	1.03 (5/486)	3.41 (17/498)	2.29 (34/1482)
SARS-CoV-2	4.82 (24/498)	0.00 (0/486)	8.43 (42/498)	4.45 (66/1482)
Human metapneumovirus	4.62 (23/498)	8.23 (40/486)	3.61 (18/498)	5.47 (81/1482)
Human rhinovirus/enterovirus	12.85 (64/498)	13.99 (68/486)	9.04 (45/498)	11.94 (177/1482)
Influenza A virus	12.65 (63/498)	8.64 (42/486)	4.82 (24/498)	8.70 (129/1482)
Influenza A virus H1	0.00 (0/498)	0.00 (0/486)	0.00 (0/498)	0.00 (0/1482)
Influenza A virus H3	12.45 (62/498)	6.38 (31/486)	4.62 (23/498)	7.83 (116/1482)
Influenza A virus H1-2009	0.20 (1/498)	2.26 (11/486)	0.20 (1/498)	0.88 (13/1482)
Influenza B virus	13.45 (67/498)	1.85 (9/486)	5.22 (26/498)	6.88 (102/1482)
Parainfluenza virus 1	1.61 (8/498)	0.00 (0/486)	0.20 (1/498)	0.61 (9/1482)
Parainfluenza virus 2	1.00 (5/498)	0.00 (0/486)	0.00 (0/498)	0.34 (5/1482)
Parainfluenza virus 3	1.00 (5/498)	0.62 (3/486)	0.40 (2/498)	0.67 (10/1482)
Parainfluenza virus 4	1.41 (7/498)	0.00 (0/486)	0.80 (4/498)	0.74 (11/1482)
Respiratory syncytial virus	12.25 (61/498)	6.58 (32/486)	13.05 (65/498)	10.66 (158/1482)
Bordetella parapertussis	0.00 (0/498)	0.00 (0/486)	0.00 (0/498)	0.00 (0/1482)
Bordetella pertussis	0.00 (0/498)	0.00 (0/486)	0.00 (0/498)	0.00 (0/1482)
Chlamydia pneumoniae	0.00 (0/498)	0.00 (0/486)	0.20 (1/498)	0.07 (1/1482)
Mycoplasma pneumoniae	0.20 (1/498)	3.91 (19/486)	0.00 (0/498)	1.35 (20/1482)

Values are percent (n positive / n tested). Detections are those reported by the investigational system and are descriptive; no statistical comparison between sites was performed.

Table S4. Combinations of organisms reported by the investigational system in the 108 prospective specimens with more than one organism detected, excluding influenza A subtyping combinations.

Analyte 1	Analyte 2	Analyte 3	Analyte 4	Specimens, n	Of which ≥ 1 organism not detected by the comparator, n	Target(s) not detected by the comparator
Adenovirus	HCoV-229E			2	0	–
Adenovirus	HCoV-HKU1	Respiratory syncytial virus		1	0	–
Adenovirus	HCoV-OC43	Human rhinovirus/enterovirus		1	0	–
Adenovirus	HCoV-OC43	Respiratory syncytial virus	SARS-CoV-2	1	0	–
Adenovirus	Human rhinovirus/enterovirus			4	0	–
Adenovirus	Human rhinovirus/enterovirus	Parainfluenza virus 3		2	0	–
Adenovirus	Human rhinovirus/enterovirus	Respiratory syncytial virus		2	1	Adenovirus (1)
Adenovirus	Influenza A virus H3			3	0	–
Adenovirus	Influenza B virus			1	1	Adenovirus (1)
Adenovirus	Respiratory syncytial virus			1	0	–
C. pneumoniae	Influenza A virus H3			1	0	–
HCoV-229E	HCoV-OC43			2	0	–
HCoV-229E	Human metapneumovirus			1	0	–
HCoV-229E	Human metapneumovirus	Human rhinovirus/enterovirus	Parainfluenza virus 1	1	1	Human rhinovirus/enterovirus (1)
HCoV-229E	Human rhinovirus/enterovirus			1	0	–
HCoV-229E	Influenza A virus H3			4	1	HCoV-229E (1)
HCoV-229E	Influenza B virus			2	0	–
HCoV-229E	Respiratory syncytial virus			2	0	–
HCoV-229E	SARS-CoV-2			1	0	–
HCoV-HKU1	Human metapneumovirus			2	0	–
HCoV-HKU1	Human rhinovirus/enterovirus			1	0	–
HCoV-HKU1	Human rhinovirus/enterovirus	M. pneumoniae		1	0	–
HCoV-HKU1	Human rhinovirus/enterovirus	Respiratory syncytial virus		1	1	HCoV-HKU1 (1)
HCoV-HKU1	Influenza A virus H1-2009			1	0	–
HCoV-HKU1	Influenza A virus H3			2	0	–
HCoV-HKU1	Influenza B virus			1	0	–
HCoV-HKU1	M. pneumoniae			1	1	M. pneumoniae (1)
HCoV-HKU1	Respiratory syncytial virus			8	1	Respiratory syncytial virus (1)
HCoV-HKU1	SARS-CoV-2			1	0	–

HCoV-NL63	Human metapneumovirus			1	0	–
HCoV-NL63	Human rhinovirus/enterovirus			1	0	–
HCoV-NL63	Influenza A virus H3			1	0	–
HCoV-NL63	Influenza B virus			1	1	HCoV-NL63 (1)
HCoV-NL63	Respiratory syncytial virus			3	0	–
HCoV-OC43	Human metapneumovirus			2	0	–
HCoV-OC43	Human metapneumovirus	Influenza A virus H1-2009		1	1	HCoV-OC43 (1)
HCoV-OC43	Parainfluenza virus 3			1	0	–
HCoV-OC43	Respiratory syncytial virus			1	0	–
HCoV-OC43	SARS-CoV-2			1	0	–
Human metapneumovirus	Human rhinovirus/enterovirus			5	3	Human rhinovirus/enterovirus (2), Human metapneumovirus (1)
Human metapneumovirus	Human rhinovirus/enterovirus	Respiratory syncytial virus	SARS-CoV-2	1	0	–
Human metapneumovirus	Influenza B virus			1	0	–
Human metapneumovirus	Parainfluenza virus 2	Respiratory syncytial virus		1	0	–
Human metapneumovirus	Respiratory syncytial virus			1	0	–
Human rhinovirus/enterovirus	Influenza A virus H3			5	0	–
Human rhinovirus/enterovirus	Influenza B virus			5	0	–
Human rhinovirus/enterovirus	M. pneumoniae			1	1	Human rhinovirus/enterovirus (1)
Human rhinovirus/enterovirus	Parainfluenza virus 1			1	0	–
Human rhinovirus/enterovirus	Parainfluenza virus 3	Respiratory syncytial virus		1	1	Parainfluenza virus 3 (1)
Human rhinovirus/enterovirus	Respiratory syncytial virus			9	0	–
Human rhinovirus/enterovirus	SARS-CoV-2			1	0	–
Influenza A virus H3	M. pneumoniae			1	0	–
Influenza A virus H3	Parainfluenza virus 1			1	0	–
Influenza A virus H3	Respiratory syncytial virus			2	0	–
Influenza B virus	Respiratory syncytial virus			4	0	–
Parainfluenza virus 4	Respiratory syncytial virus			2	1	Parainfluenza virus 4 (1)
Respiratory syncytial virus	SARS-CoV-2			1	0	–
**Two organisms**				**94**	**10**	
**Three organisms**				**11**	**4**	
**Four organisms**				**3**	**1**	
**All specimens with more than one organism**				**108**	**15**	

Row counts sum to the stated totals (94 + 11 + 3 = 108 specimens; 10 + 4 + 1 = 15 specimens with at least one organism not detected by the comparator). The sixth column counts specimens in which the investigational system reported at least one organism the comparator did not detect; this is disagreement relative to the comparator and does not establish which method was correct.

Table S5. Multiple-organism combinations reported by the comparator method in which the investigational system did not detect at least one organism in at least one specimen.

Analyte 1	Analyte 2	Analyte 3	Analyte 4	Comparator specimens with this combination, n	Of which ≥ 1 organism not detected by the investigational system, n	Target(s) not detected by the investigational system
Adenovirus	HCoV-HKU1	Influenza A virus	Respiratory syncytial virus	1	1	Influenza A virus (1)
Adenovirus	Human rhinovirus/enterovirus			6	2	Human rhinovirus/enterovirus (1), Adenovirus (1)
HCoV-HKU1	Human metapneumovirus	Human rhinovirus/enterovirus		1	1	Human rhinovirus/enterovirus (1)
HCoV-HKU1	Influenza A virus H3			3	1	Influenza A virus (1), Influenza A virus H3 (1)
HCoV-HKU1	SARS-CoV-2			2	1	SARS-CoV-2 (1)
HCoV-OC43	Influenza A virus			1	1	Influenza A virus (1)
HCoV-OC43	Influenza B virus	SARS-CoV-2		1	1	Influenza B virus (1)
Human rhinovirus/enterovirus	Influenza A virus			2	2	Influenza A virus (2)
Human rhinovirus/enterovirus	Influenza A virus H3			6	1	Human rhinovirus/enterovirus (1)
Human rhinovirus/enterovirus	Parainfluenza virus 1			2	1	Human rhinovirus/enterovirus (1), Parainfluenza virus 1 (1)
Human rhinovirus/enterovirus	Respiratory syncytial virus			13	1	Human rhinovirus/enterovirus (1)
Influenza A virus H3	Respiratory syncytial virus			4	2	Respiratory syncytial virus (2)
**Two organisms**				**39**	**12**	
**Three organisms**				**2**	**2**	
**Four organisms**				**1**	**1**	
**All listed combinations**				**42**	**15**	

Only comparator combinations in which at least one specimen was not reproduced in full by the investigational system are listed. The fifth column gives the number of prospective specimens in which the comparator reported each combination (39 + 2 + 1 = 42); the sixth column gives the number of those specimens in which the investigational system did not detect at least one of the organisms (12 + 2 + 1 = 15). In the remaining 27 specimens the investigational system reproduced the combination in full. Comparator combinations that the investigational system reproduced in full in every specimen are not listed. The 15 specimens counted here refer to the opposite direction of disagreement from the 15 counted in Table S4: those are specimens in which the investigational system reported an organism the comparator did not detect, whereas these are specimens in which the investigational system did not detect an organism the comparator reported. Numbers in parentheses are the count of specimens affected for that target. Unsubtyped influenza A is reported as influenza A virus.

Table S6. Results of the third molecular assay in specimens with discordant results, by target and direction of discordance.

Target	Specimen set	Direction of discordance	Discordant specimens, n	Third-assay result matched the comparator, n	Third-assay result matched the investigational system, n
Adenovirus	Prospective	Inv−/Comp+	2	1	1
Adenovirus	Prospective	Inv+/Comp−	2	2	0
HCoV-229E	Prospective	Inv+/Comp−	2	2	0
HCoV-HKU1	Prospective	Inv−/Comp+	1	0	1
HCoV-HKU1	Prospective	Inv+/Comp−	2	1	1
HCoV-NL63	Prospective	Inv+/Comp−	1	1	0
HCoV-OC43	Prospective	Inv+/Comp−	3	1	2
SARS-CoV-2	Prospective	Inv−/Comp+	2	0	2
SARS-CoV-2	Prospective	Inv+/Comp−	1	1	0
Human metapneumovirus	Prospective	Inv+/Comp−	2	1	1
Human rhinovirus/enterovirus	Prospective	Inv−/Comp+	16	3	13
Human rhinovirus/enterovirus	Prospective	Inv+/Comp−	5	3	2
Influenza A virus	Prospective	Inv−/Comp+	5	1	4
Influenza A virus H3	Prospective	Inv−/Comp+	1	0	1
Influenza B virus	Prospective	Inv−/Comp+	2	1	1
Influenza B virus	Prospective	Inv+/Comp−	1	0	1
Parainfluenza virus 1	Prospective	Inv−/Comp+	1	0	1
Parainfluenza virus 3	Prospective	Inv+/Comp−	3	0	3
Parainfluenza virus 4	Prospective	Inv+/Comp−	3	1	2
Respiratory syncytial virus	Prospective	Inv−/Comp+	4	2	2
Respiratory syncytial virus	Prospective	Inv+/Comp−	5	4	1
Mycoplasma pneumoniae	Prospective	Inv−/Comp+	1	1	0
Mycoplasma pneumoniae	Prospective	Inv+/Comp−	3	0	3
Adenovirus	Archived	Inv−/Comp+	3	0	3
Adenovirus	Archived	Inv+/Comp−	1	1	0
SARS-CoV-2	Archived	Inv+/Comp−	1	1	0
Human metapneumovirus	Archived	Inv−/Comp+	2	0	2
Human rhinovirus/enterovirus	Archived	Inv−/Comp+	3	2	1
Human rhinovirus/enterovirus	Archived	Inv+/Comp−	1	0	1
Parainfluenza virus 3	Archived	Inv+/Comp−	1	0	1
Parainfluenza virus 4	Archived	Inv−/Comp+	1	0	1
Parainfluenza virus 4	Archived	Inv+/Comp−	1	0	1
Bordetella pertussis	Archived	Inv+/Comp−	1	0	1
Mycoplasma pneumoniae	Archived	Inv−/Comp+	1	0	1
Mycoplasma pneumoniae	Archived	Inv+/Comp−	2	1	1
**All targets**	**Both sets**	**Both directions**	**86**	**31**	**55**

Inv−/Comp+, investigational system negative and comparator positive (counted as false negative relative to the comparator); Inv+/Comp−, the converse (counted as false positive relative to the comparator). For Inv−/Comp+ discordances, a match with the comparator means the third assay also detected the target; for Inv+/Comp− discordances, it means the third assay also failed to detect it. The third assay was applied only to discordant specimens and has its own target design and analytical sensitivity; its results are descriptive and do not establish which method was correct. Discordance counts in this table reconcile exactly with the numerators and denominators in Table 3 and Table 4 of the main text. Adjudication results were not used to reclassify results in the primary analysis, which retains the comparator as the reference throughout.

Table S7. Completed STARD 2015 checklist for reporting studies of diagnostic accuracy, with the location of each item in the manuscript. Section numbers refer to the main text.

No.	STARD 2015 item	Reported in
	**Title or abstract**	
1	Identification as a study of diagnostic accuracy using at least one measure of accuracy	Title; Abstract (positive and negative percent agreement)
	**Abstract**	
2	Structured summary of study design, methods, results and conclusions	Abstract
	**Introduction**	
3	Scientific and clinical background, including the intended use and clinical role of the index test	Section 1
4	Study objectives and hypotheses	Section 1, final paragraph; Section 2.1 (primary objective, prespecified acceptance criteria; not an equivalence or non-inferiority design)
	**Methods: study design**	
5	Whether data collection was planned before the index test and reference standard were performed (prospective) or after (retrospective)	Section 2.1 and Section 2.2 (prospective); Section 2.6 (archived specimens selected on historical result)
	**Methods: participants**	
6	Eligibility criteria	Section 2.2
7	On what basis potentially eligible participants were identified	Section 2.2 (provider-suspected respiratory infection; listed symptoms)
8	Where and when potentially eligible participants were identified (setting, location and dates)	Section 2.2; Table 1
9	Whether participants formed a consecutive, random or convenience series	Section 2.1 and Section 2.2 (all-comers; consecutive residual specimens)
	**Methods: test methods**	
10a	Index test, in sufficient detail to allow replication	Section 2.3
10b	Reference standard, in sufficient detail to allow replication	Section 2.4
11	Rationale for choosing the reference standard (if alternatives exist)	Section 1; Section 2.4 (cleared multiplex panel in routine use); rationale for not using a composite standard, Section 4.1
12a	Definition of and rationale for test positivity cut-offs of the index test, distinguishing prespecified from exploratory	Section 2.3 (qualitative detected/not-detected calls per the manufacturer's instructions; prespecified)
12b	Definition of and rationale for test positivity cut-offs of the reference standard, distinguishing prespecified from exploratory	Section 2.4 (qualitative calls per the manufacturer's instructions; prespecified)
13a	Whether clinical information and reference standard results were available to the performers or readers of the index test	Sections 2.4 and 2.5 (mutually blinded; personnel restricted to their own laboratory's data; laboratory assignment, specimen coding and EDC access)
13b	Whether clinical information and index test results were available to the assessors of the reference standard	Sections 2.4 and 2.5 (mutually blinded); Section 2.4 (adjudicating laboratory blinded to original results)
	**Methods: analysis**	
14	Methods for estimating or comparing measures of diagnostic accuracy	Section 2.8
15	How indeterminate index test or reference standard results were handled	Section 2.8; Section 3.2; Figure 1; Table S9
16	How missing data on the index test and reference standard were handled	Section 2.8 (specimens without a paired valid result excluded and reported separately); Figure 1
17	Any analyses of variability in diagnostic accuracy, distinguishing prespecified from exploratory	Prespecified: fresh versus frozen categories, Table S1. Exploratory and descriptive only: by site (Table S3) and by age stratum (Figure 2); no statistical comparison was made (Section 2.8)
18	Intended sample size and how it was determined	Section 2.9 (calculated by the method of Buderer; target enrolment

		approximately 1500)
	**Results: participants**	
19	Flow of participants, using a diagram	Figure 1
20	Baseline demographic and clinical characteristics of participants	Table 2; Table S2 (archived set)
21a	Distribution of severity of disease in those with the target condition	Not reported; addressed as a limitation in Section 4.1
21b	Distribution of alternative diagnoses in those without the target condition	Not reported; addressed as a limitation in Section 4.1. Co-detections among test-positive specimens are given in Section 3.6 and Tables S4 and S5
22	Time interval and any clinical interventions between index test and reference standard	Section 2.2 (aliquots of a single specimen; fresh specimens tested within 72 h of collection, frozen specimens within the specified storage window); Section 2.4 (parallel testing). No clinical intervention occurred between the two tests
	**Results: test results**	
23	Cross tabulation of the index test results by the results of the reference standard	Tables 3, 4 and 5; Table S1 (by specimen category)
24	Estimates of diagnostic accuracy and their precision (such as 95% confidence intervals)	Tables 3, 4, 5 and 6; Figure 3
25	Any adverse events from performing the index test or the reference standard	Section 2.7 (none reported)
	**Discussion**	
26	Study limitations, including sources of potential bias, statistical uncertainty and generalisability	Section 4.1
27	Implications for practice, including the intended use and clinical role of the index test	Section 4; Section 5
	**Other information**	
28	Registration number and name of registry	Section 2.1 (non-interventional; not registered)
29	Where the full study protocol can be accessed	Section 2.1 (available from the corresponding authors on reasonable request)
30	Sources of funding and other support; role of funders	Funding; Conflicts of Interest

Items marked “Not reported” were not addressed in this report. Item 18: the sample-size calculation and its assumptions are stated in Section 2.9. Items 21a and 21b: clinical severity and final clinical diagnoses were outside the scope of this method-comparison study and were not collected; this is stated as a limitation in Section 4.1. Checklist adapted from Bossuyt et al., BMJ 2015;351:h5527 [19].

Table S8. Strains, limits of detection and concentrations used to prepare the contrived specimens (copies/mL).

Target	Strain and source	LoD	2× LoD	5× LoD	100× LoD
Influenza A virus H1	A/New Caledonia/20/1999 (H1N1); BEI Resources NR-41799	1,666.67	3,333.34	8,333.35	166,667
Bordetella parapertussis	Strain 508 and 344 (NCTC 10853); ATCC 15237	500	1,000	2,500	50,000
Bordetella pertussis	Strain H973; BEI Resources NR-42460	500	1,000	2,500	50,000
Chlamydia pneumoniae	Strain TW-183; ATCC VR-2282	500	1,000	2,500	50,000

LoD, limit of detection. Information from the sponsor's LoD validation and specimen preparation records. The same strains were used for LoD determination and for contrived-specimen preparation. LoD was established on the investigational system by testing tenfold serial dilutions in triplicate, followed by confirmatory testing of 20 replicates at concentrations near the estimated LoD; the LoD was the lowest concentration giving at least 95% positive results, a result being positive when the predefined target-specific cycle threshold and TraceR acceptance criteria were met. The matrix was pooled negative clinical nasopharyngeal specimens confirmed negative with FilmArray RP2.1 before spiking. For each target and concentration, a common spiked stock was divided into separate aliquots (30 at 2× LoD, 10 at 5× LoD and 10 at 100× LoD), each processed and tested individually.

Table S9. Initial and final invalid results and repeat testing in the 1485 prospective specimens tested.

Measure	Investigational system	Comparator
Specimens tested (first test)	1485	1485
Initial invalid specimens, n (%)	10 (0.67)	11 (0.74)
Specimens retested, n	10	11
Additional specimen tests, n	10	11
Instrument runs containing retests, n	7	11
Specimens valid on retesting, n	9	9
Final invalid specimens, n (%)	1 (0.07)	2 (0.13)
Run-level failures affecting more than one specimen, n	0	0

An initial invalid result is an invalid result on the first test of a specimen; a final invalid result is a specimen that remained invalid after repeat testing. Each initially invalid specimen was retested once, and the three final invalid specimens were excluded from the agreement analysis. Investigational testing comprised 269 instrument runs, excluding quality-control-only runs; 9 runs (3.35%) contained at least one invalid specimen result, and when more than one specimen in a run was invalid the remaining specimens were unaffected. Invalid investigational results occurred in runs by four of the ten operators (invalid test results/runs containing invalid results/total runs: 4/4/29, 1/1/35, 5/3/84 and 1/1/26); no invalid results were recorded for the other six operators, who performed the remaining 95 runs (individual counts not available). Review of the invalid tests did not identify a documented operator error. Archived (240/240) and contrived (200/200) specimens all gave valid results.
