# Supplementary material for: Clinical Performance of a Fully Integrated Sample-to-Answer Multiplex PCR Respiratory Panel: A Multicenter Evaluation Using Prospective, Archived and Contrived Nasopharyngeal Specimens
Source: Diagnostics (Basel). 2026 Oct 28;16(21):3541. doi: 10.3390/diagnostics16213541 (PMC13761482; doi:10.3390/diagnostics16213541)
Supplement: Supplementary file 1 [file diagnostics-16-03541-s001.zip › diagnostics-4604166_FileS1.pdf]

Supplementary File S1. Analysis code for the agreement estimates and confidence intervals

Clinical Performance of a Fully Integrated Sample-to-Answer Multiplex PCR Respiratory Panel: A Multicenter Evaluation Using Prospective, Archived and Contrived Nasopharyngeal Specimens

Purpose

This Python script recalculates the positive and negative percent agreement (PPA, NPA) with two-sided 95% Wilson score confidence intervals for the prospective (Table 3), archived (Table 4) and contrived (Table 5) specimen sets, and classifies each target against the prespecified prospective acceptance criteria (Table 6). The input is the per-target 2 × 2 counts reported in the clinical study report, which are written into the script. TP, FN, TN and FP are defined relative to the comparator for the prospective and archived sets and relative to known composition for the contrived set.

How to use

Section A lists the complete code and Section B the output it produces, so the results can be checked against Tables 3–6 without running the script. To run it, copy the code in Section A into a text file named `diagnostics-4604166_FileS1.py`, or use the .py file provided with this document, and run `python diagnostics-4604166_FileS1.py`. The script requires Python 3.8 or later and uses only the standard library. The output in Section B was produced with Python 3.13; every value matches the corresponding entry in Tables 3–6 of the main text.

A. Source code

```
"""
Supplementary File S1. Analysis code for diagnostics-4604166.

Recalculates positive and negative percent agreement (PPA, NPA) with two-sided
95% Wilson score confidence intervals for the prospective (Table 3), archived
(Table 4) and contrived (Table 5) specimen sets, and the per-target
classification against the prespecified acceptance criteria (Table 6).

Input: per-target 2x2 counts of the investigational system against the
comparator (prospective and archived sets) or against known composition
(contrived set), as reported in the clinical study report.
TP, FN, TN and FP are defined relative to the reference used for each set.

Requires Python 3.8 or later; standard library only.
Usage:  python diagnostics-4604166_FileS1.py
"""
from math import sqrt

Z = 1.959964  # two-sided 95%


def wilson(x, n, z=Z):
    """Return (point, lower, upper) in percent, or None when n == 0."""
    if n == 0:
        return None
    p = x / n
    denom = 1 + z * z / n
    centre = (p + z * z / (2 * n)) / denom
    half = z * sqrt(p * (1 - p) / n + z * z / (4 * n * n)) / denom
    return 100 * p, 100 * (centre - half), 100 * (centre + half)


def fmt(x, n):
    w = wilson(x, n)
    return "NE" if w is None else f"{w[0]:.1f} ({w[1]:.1f}-{w[2]:.1f})"


# target: (TP, FN, TN, FP)
PROSPECTIVE = {
    "Adenovirus": (35, 2, 1443, 2),
    "HCoV-229E": (49, 0, 1431, 2),
    "HCoV-HKU1": (59, 1, 1420, 2),
    "HCoV-NL63": (42, 0, 1439, 1),
    "HCoV-OC43": (31, 0, 1448, 3),
    "SARS-CoV-2": (65, 2, 1414, 1),
    "Human metapneumovirus": (79, 0, 1401, 2),
    "Human rhinovirus/enterovirus": (172, 16, 1289, 5),
    "Influenza A virus": (129, 5, 1348, 0),
    "Influenza A virus H1": (0, 0, 1482, 0),
    "Influenza A virus H3": (116, 1, 1365, 0),
    "Influenza A virus H1-2009": (13, 0, 1469, 0),
    "Influenza B virus": (101, 2, 1378, 1),
    "Parainfluenza virus 1": (9, 1, 1472, 0),
```

```
    "Parainfluenza virus 2": (5, 0, 1477, 0),
    "Parainfluenza virus 3": (7, 0, 1472, 3),
    "Parainfluenza virus 4": (8, 0, 1471, 3),
    "Respiratory syncytial virus": (153, 4, 1320, 5),
    "Bordetella parapertussis": (0, 0, 1482, 0),
    "Bordetella pertussis": (0, 0, 1482, 0),
    "Chlamydia pneumoniae": (1, 0, 1481, 0),
    "Mycoplasma pneumoniae": (17, 1, 1461, 3),
}

ARCHIVED = {
    "Adenovirus": (24, 3, 212, 1),
    "HCoV-229E": (1, 0, 239, 0),
    "HCoV-HKU1": (6, 0, 234, 0),
    "HCoV-NL63": (0, 0, 240, 0),
    "HCoV-OC43": (2, 0, 238, 0),
    "SARS-CoV-2": (0, 0, 239, 1),
    "Human metapneumovirus": (2, 2, 236, 0),
    "Human rhinovirus/enterovirus": (22, 3, 214, 1),
    "Influenza A virus": (32, 0, 208, 0),
    "Influenza A virus H1": (0, 0, 240, 0),
    "Influenza A virus H3": (0, 0, 240, 0),
    "Influenza A virus H1-2009": (32, 0, 208, 0),
    "Influenza B virus": (1, 0, 239, 0),
    "Parainfluenza virus 1": (36, 0, 204, 0),
    "Parainfluenza virus 2": (31, 0, 209, 0),
    "Parainfluenza virus 3": (30, 0, 209, 1),
    "Parainfluenza virus 4": (28, 1, 210, 1),
    "Respiratory syncytial virus": (2, 0, 238, 0),
    "Bordetella parapertussis": (0, 0, 240, 0),
    "Bordetella pertussis": (1, 0, 238, 1),
    "Chlamydia pneumoniae": (4, 0, 236, 0),
    "Mycoplasma pneumoniae": (27, 1, 210, 2),
}

# contrived: target -> {concentration: (TP, FN, TN, FP)}; reference = known composition
CONTRIVED = {
    t: {"2x LoD": (30, 0, 90, 0), "5x LoD": (10, 0, 30, 0), "100x LoD": (10, 0, 30, 0)}
    for t in ["Influenza A virus H1", "Bordetella parapertussis", "Bordetella pertussis", "Chlamydia pneumoniae"]
}


def criteria(target, tp, fn):
    """Prespecified prospective PPA criterion: point >= 90% (95% for SARS-CoV-2) and lower bound >= 80%."""
    w = wilson(tp, tp + fn)
    if w is None:
        return "NE (no positives)"
    threshold = 95 if target == "SARS-CoV-2" else 90
    return "Met" if (w[0] >= threshold and w[1] >= 80) else "Not met (lower bound < 80%)"


def table(title, data):
    print(f"\n{title}")
    print(f"{'Target':30s} {'TP/(TP+FN)':>12s} {'PPA % (95% CI)':>22s} {'TN/(TN+FP)':>12s} {'NPA % (95% CI)':>22s}")
    for t, (tp, fn, tn, fp) in data.items():
        print(f"{t:30s} {f'{tp}/{tp+fn}':>12s} {fmt(tp, tp+fn):>22s} {f'{tn}/{tn+fp}':>12s} {fmt(tn, tn+fp):>22s}")


if __name__ == "__main__":
    table("Table 3. Prospective specimens (n = 1482)", PROSPECTIVE)
    table("Table 4. Archived specimens (n = 240)", ARCHIVED)
    print("\nTable 5. Contrived specimens (reference: known composition)")
    for t, rows in CONTRIVED.items():
        tot = [sum(v[i] for v in rows.values()) for i in range(4)]
        for conc, (tp, fn, tn, fp) in list(rows.items()) + [("Combined", tuple(tot))]:
            print(f"{t:30s} {conc:>9s} {f'{tp}/{tp+fn}':>8s} {fmt(tp, tp+fn):>22s} {f'{tn}/{tn+fp}':>8s} {fmt(tn, tn+fp):>22s}")
    print("\nTable 6. Classification against the prospective acceptance criteria (no pooling across sets)")
    counts = {"Met": 0, "Not met (lower bound < 80%)": 0, "NE (no positives)": 0}
    for t, (tp, fn, tn, fp) in PROSPECTIVE.items():
        c = criteria(t, tp, fn)
        counts[c] += 1
        a = ARCHIVED[t]
        arch = f"{a[0]}/{a[0]+a[1]}" if a[0] + a[1] else "0/0 (NE)"
        con = "50/50" if t in CONTRIVED else "-"
        print(f"{t:30s} {f'{tp}/{tp+fn}':>9s} {fmt(tp, tp+fn):>22s}  {c:28s} {arch:>10s} {con:>6s}")
    print("\nSummary:", counts)
    npa_ok = all(wilson(tn, tn + fp)[0] >= 95 and wilson(tn, tn + fp)[1] >= 80 for tp, fn, tn, fp in PROSPECTIVE.values())
    print("NPA criterion met for all prospective targets:", npa_ok)
```

B. Output (Python 3.13)

Table 3. Prospective specimens (n = 1482)

Target	TP/(TP+FN)	PPA % (95% CI)	TN/(TN+FP)	NPA % (95% CI)
Adenovirus	35/37	94.6 (82.3-98.5)	1443/1445	99.9 (99.5-100.0)
HCoV-229E	49/49	100.0 (92.7-100.0)	1431/1433	99.9 (99.5-100.0)
HCoV-HKU1	59/60	98.3 (91.1-99.7)	1420/1422	99.9 (99.5-100.0)
HCoV-NL63	42/42	100.0 (91.6-100.0)	1439/1440	99.9 (99.6-100.0)
HCoV-OC43	31/31	100.0 (89.0-100.0)	1448/1451	99.8 (99.4-99.9)
SARS-CoV-2	65/67	97.0 (89.8-99.2)	1414/1415	99.9 (99.6-100.0)
Human metapneumovirus	79/79	100.0 (95.4-100.0)	1401/1403	99.9 (99.5-100.0)
Human rhinovirus/enterovirus	172/188	91.5 (86.6-94.7)	1289/1294	99.6 (99.1-99.8)
Influenza A virus	129/134	96.3 (91.6-98.4)	1348/1348	100.0 (99.7-100.0)
Influenza A virus H1	0/0	NE	1482/1482	100.0 (99.7-100.0)
Influenza A virus H3	116/117	99.1 (95.3-99.8)	1365/1365	100.0 (99.7-100.0)
Influenza A virus H1-2009	13/13	100.0 (77.2-100.0)	1469/1469	100.0 (99.7-100.0)
Influenza B virus	101/103	98.1 (93.2-99.5)	1378/1379	99.9 (99.6-100.0)
Parainfluenza virus 1	9/10	90.0 (59.6-98.2)	1472/1472	100.0 (99.7-100.0)
Parainfluenza virus 2	5/5	100.0 (56.6-100.0)	1477/1477	100.0 (99.7-100.0)
Parainfluenza virus 3	7/7	100.0 (64.6-100.0)	1472/1475	99.8 (99.4-99.9)
Parainfluenza virus 4	8/8	100.0 (67.6-100.0)	1471/1474	99.8 (99.4-99.9)
Respiratory syncytial virus	153/157	97.5 (93.6-99.0)	1320/1325	99.6 (99.1-99.8)
Bordetella parapertussis	0/0	NE	1482/1482	100.0 (99.7-100.0)
Bordetella pertussis	0/0	NE	1482/1482	100.0 (99.7-100.0)
Chlamydia pneumoniae	1/1	100.0 (20.7-100.0)	1481/1481	100.0 (99.7-100.0)
Mycoplasma pneumoniae	17/18	94.4 (74.2-99.0)	1461/1464	99.8 (99.4-99.9)

Table 4. Archived specimens (n = 240)

Target	TP/(TP+FN)	PPA % (95% CI)	TN/(TN+FP)	NPA % (95% CI)
Adenovirus	24/27	88.9 (71.9-96.1)	212/213	99.5 (97.4-99.9)
HCoV-229E	1/1	100.0 (20.7-100.0)	239/239	100.0 (98.4-100.0)
HCoV-HKU1	6/6	100.0 (61.0-100.0)	234/234	100.0 (98.4-100.0)
HCoV-NL63	0/0	NE	240/240	100.0 (98.4-100.0)
HCoV-OC43	2/2	100.0 (34.2-100.0)	238/238	100.0 (98.4-100.0)
SARS-CoV-2	0/0	NE	239/240	99.6 (97.7-99.9)
Human metapneumovirus	2/4	50.0 (15.0-85.0)	236/236	100.0 (98.4-100.0)
Human rhinovirus/enterovirus	22/25	88.0 (70.0-95.8)	214/215	99.5 (97.4-99.9)
Influenza A virus	32/32	100.0 (89.3-100.0)	208/208	100.0 (98.2-100.0)
Influenza A virus H1	0/0	NE	240/240	100.0 (98.4-100.0)
Influenza A virus H3	0/0	NE	240/240	100.0 (98.4-100.0)
Influenza A virus H1-2009	32/32	100.0 (89.3-100.0)	208/208	100.0 (98.2-100.0)
Influenza B virus	1/1	100.0 (20.7-100.0)	239/239	100.0 (98.4-100.0)
Parainfluenza virus 1	36/36	100.0 (90.4-100.0)	204/204	100.0 (98.2-100.0)
Parainfluenza virus 2	31/31	100.0 (89.0-100.0)	209/209	100.0 (98.2-100.0)
Parainfluenza virus 3	30/30	100.0 (88.6-100.0)	209/210	99.5 (97.4-99.9)
Parainfluenza virus 4	28/29	96.6 (82.8-99.4)	210/211	99.5 (97.4-99.9)
Respiratory syncytial virus	2/2	100.0 (34.2-100.0)	238/238	100.0 (98.4-100.0)
Bordetella parapertussis	0/0	NE	240/240	100.0 (98.4-100.0)
Bordetella pertussis	1/1	100.0 (20.7-100.0)	238/239	99.6 (97.7-99.9)
Chlamydia pneumoniae	4/4	100.0 (51.0-100.0)	236/236	100.0 (98.4-100.0)
Mycoplasma pneumoniae	27/28	96.4 (82.3-99.4)	210/212	99.1 (96.6-99.7)

Table 5. Contrived specimens (reference: known composition)

Influenza A virus H1	2x LoD	30/30	100.0 (88.6-100.0)	90/90	100.0 (95.9-100.0)
Influenza A virus H1	5x LoD	10/10	100.0 (72.2-100.0)	30/30	100.0 (88.6-100.0)
Influenza A virus H1	100x LoD	10/10	100.0 (72.2-100.0)	30/30	100.0 (88.6-100.0)
Influenza A virus H1	Combined	50/50	100.0 (92.9-100.0)	150/150	100.0 (97.5-100.0)
Bordetella parapertussis	2x LoD	30/30	100.0 (88.6-100.0)	90/90	100.0 (95.9-100.0)
Bordetella parapertussis	5x LoD	10/10	100.0 (72.2-100.0)	30/30	100.0 (88.6-100.0)
Bordetella parapertussis	100x LoD	10/10	100.0 (72.2-100.0)	30/30	100.0 (88.6-100.0)
Bordetella parapertussis	Combined	50/50	100.0 (92.9-100.0)	150/150	100.0 (97.5-100.0)
Bordetella pertussis	2x LoD	30/30	100.0 (88.6-100.0)	90/90	100.0 (95.9-100.0)
Bordetella pertussis	5x LoD	10/10	100.0 (72.2-100.0)	30/30	100.0 (88.6-100.0)
Bordetella pertussis	100x LoD	10/10	100.0 (72.2-100.0)	30/30	100.0 (88.6-100.0)
Bordetella pertussis	Combined	50/50	100.0 (92.9-100.0)	150/150	100.0 (97.5-100.0)
Chlamydia pneumoniae	2x LoD	30/30	100.0 (88.6-100.0)	90/90	100.0 (95.9-100.0)
Chlamydia pneumoniae	5x LoD	10/10	100.0 (72.2-100.0)	30/30	100.0 (88.6-100.0)
Chlamydia pneumoniae	100x LoD	10/10	100.0 (72.2-100.0)	30/30	100.0 (88.6-100.0)
Chlamydia pneumoniae	Combined	50/50	100.0 (92.9-100.0)	150/150	100.0 (97.5-100.0)

Table 6. Classification against the prospective acceptance criteria (no pooling across sets)

Adenovirus	35/37	94.6 (82.3-98.5)	Met	24/27	-
HCoV-229E	49/49	100.0 (92.7-100.0)	Met	1/1	-
HCoV-HKU1	59/60	98.3 (91.1-99.7)	Met	6/6	-
HCoV-NL63	42/42	100.0 (91.6-100.0)	Met	0/0 (NE)	-
HCoV-OC43	31/31	100.0 (89.0-100.0)	Met	2/2	-
SARS-CoV-2	65/67	97.0 (89.8-99.2)	Met	0/0 (NE)	-
Human metapneumovirus	79/79	100.0 (95.4-100.0)	Met	2/4	-
Human rhinovirus/enterovirus	172/188	91.5 (86.6-94.7)	Met	22/25	-
Influenza A virus	129/134	96.3 (91.6-98.4)	Met	32/32	-
Influenza A virus H1	0/0	NE	NE (no positives)	0/0 (NE)	50/50
Influenza A virus H3	116/117	99.1 (95.3-99.8)	Met	0/0 (NE)	-
Influenza A virus H1-2009	13/13	100.0 (77.2-100.0)	Not met (lower bound < 80%)	32/32	-
Influenza B virus	101/103	98.1 (93.2-99.5)	Met	1/1	-
Parainfluenza virus 1	9/10	90.0 (59.6-98.2)	Not met (lower bound < 80%)	36/36	-
Parainfluenza virus 2	5/5	100.0 (56.6-100.0)	Not met (lower bound < 80%)	31/31	-
Parainfluenza virus 3	7/7	100.0 (64.6-100.0)	Not met (lower bound < 80%)	30/30	-
Parainfluenza virus 4	8/8	100.0 (67.6-100.0)	Not met (lower bound < 80%)	28/29	-
Respiratory syncytial virus	153/157	97.5 (93.6-99.0)	Met	2/2	-
Bordetella parapertussis	0/0	NE	NE (no positives)	0/0 (NE)	50/50

Bordetella pertussis	0/0	NE	NE (no positives)	1/1	50/50
Chlamydia pneumoniae	1/1	100.0 (20.7-100.0)	Not met (lower bound < 80%)	4/4	50/50
Mycoplasma pneumoniae	17/18	94.4 (74.2-99.0)	Not met (lower bound < 80%)	27/28	-

Summary: {'Met': 12, 'Not met (lower bound < 80%)': 7, 'NE (no positives)': 3}
NPA criterion met for all prospective targets: True
